# α-synuclein buildup is alleviated *via* ESCRT-dependent endosomal degradation brought about by p38MAPK inhibition in cells expressing p25α

**DOI:** 10.1016/j.jbc.2022.102531

**Published:** 2022-09-24

**Authors:** Helena Borland, Izabela Rasmussen, Kaare Bjerregaard-Andersen, Michel Rasmussen, Anders Olsen, Frederik Vilhardt

**Affiliations:** 1 Department of Cellular and Molecular Medicine, The Faculty of Health Sciences, The Panum Institute, University of Copenhagen, Copenhagen, Denmark; 2 Department of Cell Biology, H. Lundbeck A/S, Valby, Denmark; 3 Department of Chemistry and Bioscience, The Faculty of Engineering and Science, University of Aalborg, Aalborg, Denmark

**Keywords:** α-synuclein, TPPP/p25α, endosomes, lysosomes, autophagy, chaperone-mediated autophagy, microautophagy, ESCRT, 3-MA, 3-methyladenine, αSyn, α-synuclein, ATG5, autophagy-related 5, CMA, chaperone-mediated autophagy, DIV, day *in vitro*, DLK, dual leucine zipper kinase, ESCRT, endosomal sorting complex required for transport, ILV, intraluminal vesicle, JNK, c-Jun N-terminal kinase, LAMP, lysosome-associated membrane protein, LDH, lactate dehydrogenase, LP, leupeptin/pepstatin, mAb, monoclonal antibody, NGF, nerve growth factor, PD, Parkinson's disease, PFA, paraformaldehyde, P/S, penicillin and streptomycin, p-Ser129, phosphorylated serine-129, RT, room temperature, TCA, trichloroacetic acid

## Abstract

α-synucleinopathy is driven by an imbalance of synthesis and degradation of α-synuclein (αSyn), causing a build up of αSyn aggregates and post-translationally modified species, which not only interfere with normal cellular metabolism but also by their secretion propagates the disease. Therefore, a better understanding of αSyn degradation pathways is needed to address α-synucleinopathy. Here, we used the nerve growth factor–differentiated catecholaminergic PC12 neuronal cell line, which was conferred α-synucleinopathy by inducible expression of αSyn and tubulin polymerization-promoting protein p25α. p25α aggregates αSyn, and imposes a partial autophagosome–lysosome block to mimic aspects of lysosomal deficiency common in neurodegenerative disease. Under basal conditions, αSyn was degraded by multiple pathways but most prominently by macroautophagy and Nedd4/Ndfip1-mediated degradation. We found that expression of p25α induced strong p38MAPK activity. Remarkably, when opposed by inhibitor SB203580 or p38MAPK shRNA knockdown, endolysosomal localization and degradation of αSyn increased, and αSyn secretion and cytotoxicity decreased. This effect was specifically dependent on Hsc70 and the endosomal sorting complex required for transport machinery, but different from classical microautophagy, as the αSyn Hsc70 binding motif was unnecessary. Furthermore, in a primary neuronal (h)-αSyn seeding model, p38MAPK inhibition decreased pathological accumulation of phosphorylated serine-129-αSyn and cytotoxicity. In conclusion, p38MAPK inhibition shifts αSyn degradation from various forms of autophagy to an endosomal sorting complex required for transport–dependent uptake mechanism, resulting in increased αSyn turnover and cell viability in p25α-expressing cells. More generally, our results suggest that under conditions of autophagolysosomal malfunction, the uninterrupted endosomal pathway offers a possibility to achieve disease-associated protein degradation.

A plenitude of neurodegenerative diseases is caused by aggregation and accumulation of endogenous nerve cell proteins, reflecting an inefficient removal of pathological protein aggregates. In the case of the second most common neurodegenerative disease, Parkinson's disease (PD), α-synuclein (αSyn) constitutes the major protein in neuronal cytoplasmic inclusions (1). A strong genetic link, showing that specific mutations (2) and αSyn gene (SNCA) dosage (3) lead to synucleinopathy, places αSyn as the major driver of pathology, even though multiple factors influence the disease mechanism. Lewy bodies and other lesions with insoluble αSyn contain almost exclusively αSyn phosphorylated on serine-129 (p-Ser129) (4), the most common post-translational modification of insoluble αSyn (5).

While the role of p-Ser129 in aggregation and fibril formation is unsettled and contextual (4, 6, 7, 8), p-Ser129 levels in the brain correlates with disease severity (9, 10), and it is often used as a readout of ongoing pathology. Tubulin-polymerization promoting protein (TPPP/p25α) is also found within inclusions of PD and Lewy body disease neurons. p25α not only promotes microtubule polymerization and organization (11, 12) but also aggregates αSyn (13) and partially inhibits autophagosome fusion with lysosomes *via* its inhibitory action toward histone deacetylase-6 (14, 15, 16, 17). As a consequence, autophagosomes are exocytosed to release αSyn species to the environment (17). Thereby, p25α expression replicates cardinal features of PD or misfolding disease in general, where direct or contingent lysosomal deficiency is omnipresent (18, 19). Secretion of toxic protein aggregates is considered to be a major mechanism of disease propagation in the brain, as unaffected nerve cells take up the aggregates, which subsequently template proteopathic conversion of wildtype αSyn to perpetuate the disease (20, 21, 22, 23).

Cellular degradation by autophagy is divided into three main subtypes: macroautophagy, chaperone-mediated autophagy (CMA), and microautophagy (19, 22, 24). Both macroautophagy and CMA have been implicated in αSyn degradation (17, 25, 26). Whereas macroautophagy relies on the indiscriminate (starvation) or specific (receptor-mediated) engulfment of cytosol/cargo by membrane formation *de novo*, both CMA and microautophagy deliver soluble substrates for degradation directly into the lumen of endolysosomes. The two latter forms both rely on cytosolic Hsc70 to recruit substrates (27), but they mechanistically differ in their requirement for lysosomal-associated membrane protein 2a (LAMP2a) or the late endosomal ESCRT (endosomal sorting complex required for transport) machinery, respectively (24, 28). Of the various forms of autophagy, microautophagy is the least described in mammalian cells (24, 29) and has not previously been tied to αSyn degradation.

However, other receptors working on the surface of endolysosomes might also be involved in αSyn turnover. In particular, the functionality of the endosomal membrane protein Ndfip1 in relation to ESCRT-mediated uptake of αSyn has never been addressed directly despite the fact that its binding partner, the E3 ubiquitin ligase Nedd4, has been identified in genetic screens as a central node in the network that regulates αSyn degradation (30).

Here, we show that pharmacological or genetic inhibition of p38MAPK in nerve cells expressing p25α and αSyn induces ESCRT-dependent degradation of αSyn to alleviate accumulation of αSyn and autophagosomes, and thereby decrease synucleinopathic toxicity, while at the same time lowering the amount of secreted αSyn. Likewise, αSyn fibril–induced phosphorylation of intracellular αSyn at ser129 was dose-dependently reduced by p38MAPK inhibition in primary neurons expressing human αSyn.

## Results

### Pharmacological inhibition of p38MAPK decreases αSyn secretion in p25α-expressing PC12 cells

We have previously shown that expression of p25α in the nerve growth factor (NGF)–differentiated catecholaminergic PC12 neuronal cell line induces unconventional secretion of αSyn aggregates because of an inhibition of autophagosome to lysosome trafficking (17). In this cell model, both p25α and αSyn are expressed from a doxycycline-inducible promotor to avoid toxicity and/or adaptation in the stock culture.

In a complementary work (F. V. and A. O., unpublished results), we find that overexpression of human p25α in the dopaminergic anterior deirids neurons of *Caenorhabditis elegans* causes age-dependent neurodegeneration. Using this transgenic model to study p25α cytopathology, we identified dual leucine zipper kinase (DLK) and p38MAPK as rescue mutations of p25α-induced toxicity. Both kinases fall into an axonal p38MAPK regeneration pathway, which runs concurrently with c-Jun N-terminal kinase (JNK) stress kinase activation in worm (31). We have previously shown that p25α expression leads to strong JNK2 activation in PC12 cells, which in turn regulates secretion (exophagy) of αSyn (32). We therefore decided to further investigate the role of the effector kinase p38MAPK with respect to αSyn degradation and secretion.

**Figure 1:**
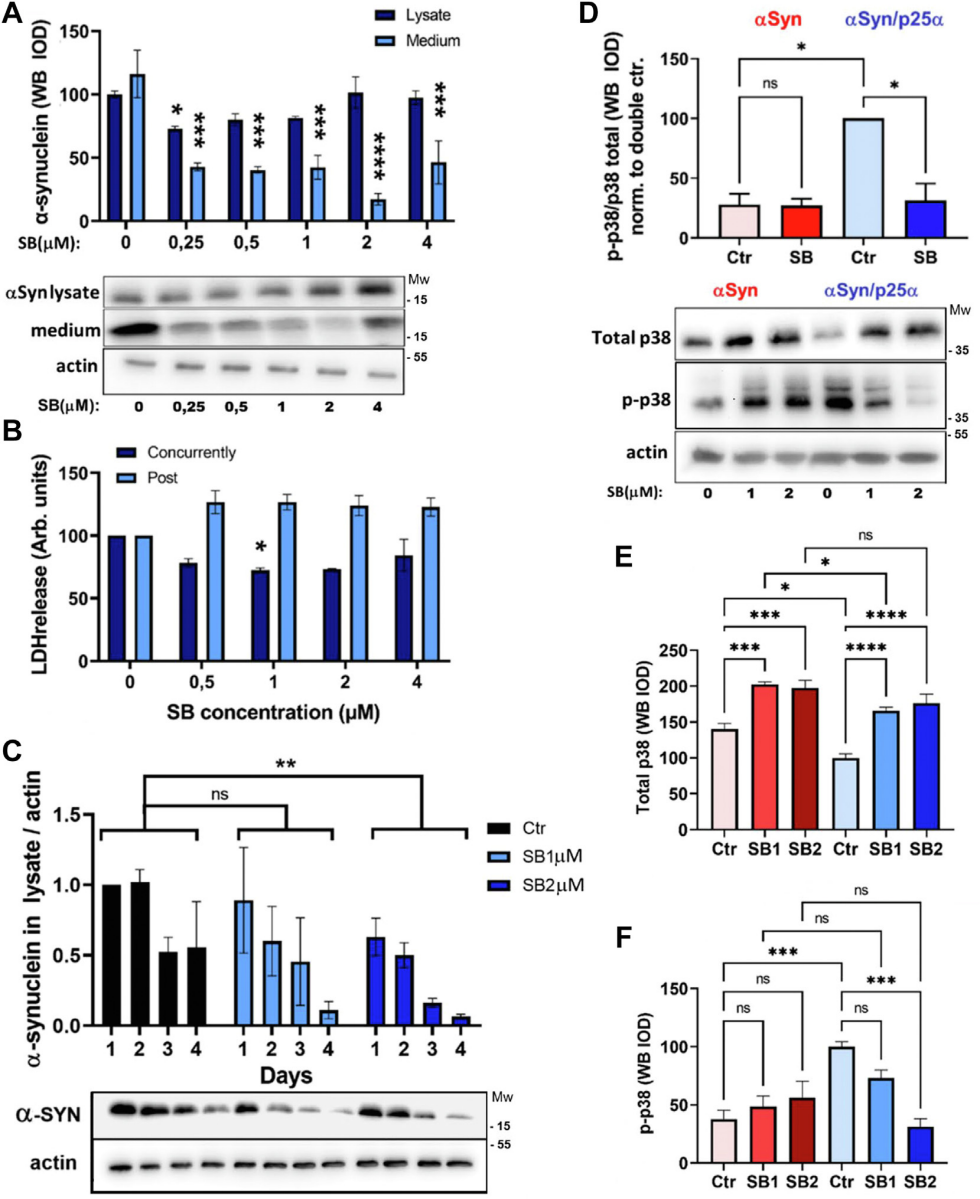
**p38MAPK inhibition by SB203580 increases αSyn turnover and lowers αSyn secretion and toxicity in cells expressing p25α.***A*, NGF-differentiated PC12-αSyn/p25α cells were treated with SB203580 as indicated with doxycycline (to induce αSyn and p25α transgenes) for 2 days before Western blot analysis of αSyn in TCA-precipitated conditioned medium and actin and αSyn in the lysate fraction (ordinary one-way ANOVA, N = 4–6). *B*, PC12-αSyn/p25α cell cultures received SB203580 either concurrently with doxycycline treatment (concurrently) or 24 h after doxycycline induction (post). After 2 days, conditioned medium was analyzed for lactate dehydrogenase (LDH) to assess cell death (Kruskal–Wallis test, N = 4). *C*, αSyn and p25α expression was induced in PC12-αSyn/p25α cells by doxycycline treatment for 24 h with/without concurrent SB203580 administration followed by doxycycline washout. Cells were chased for a further 4 days with/without SB203580 with sampling of replicate wells each day for analysis of intracellular αSyn by Western blotting. The Western blot shows a representative experiment, whereas graphs represent αSyn normalized to actin Western blot band absorbance relative to D1 values (two-way ANOVA, N = 3). *D*, NGF-differentiated PC12 cells expressing αSyn or αSyn/p25α were treated or not with SB203580 at 1 or 2 μM for 2 days of transgene expression and then lysates were analyzed by Western blotting for total and phosphorylated p38MAPK and actin. The graphs show mean integrated absorbance of Western blot bands for the (*D*) ratio of phosphorylated to total p38MAPK (*i.e.*, specific activity) for a SB203580 concentration of 2 μM, the ratio being derived from the absorbances of (*E*) total p38MAPK and (*F*) p-p38MAPK bands (Kruskal–Wallis test, N = 4). All graphs show mean ± SEM. αSyn, α-synuclein; NGF, nerve growth factor; TCA, trichloroacetic acid.

NGF-differentiated PC12 cells were treated with doxycycline to induce expression of αSyn and p25α, and at the same time, administered the p38MAPK inhibitor SB203508. Two days after induction, cells were lysed and conditioned medium collected for trichloroacetic acid (TCA) precipitation of secreted αSyn. Remarkably, secretion of αSyn was significantly decreased (by up to ca. 70%) by p38MAPK inhibitor SB203580 in αSyn/p25α-expressing cells at all concentrations of SB203580 tested (0.25–4 μM) (Fig. 1*A*), which are substantially lower than the commonly used 10 to 20 μM. SB203580 also decreased cellular levels of αSyn, but under the conditions of the experiment, SB203580 failed to reveal statistically significant differences between cellular levels of αSyn at all concentrations except 0.25 μM, because of the difficulty in observing modest declines in intracellular αSyn levels in cells with a high αSyn expression driven by the strong TetO promotor. These changes correlated with a significantly lowered cytotoxicity (as measured by lactate dehydrogenase (LDH)release) in cells treated with SB203580 at 1 μM (Fig. 1*B*). Notably, SB203580 only had a beneficial effect when added concurrently with doxycycline induction of αSyn/p25α expression. Addition of inhibitor 24 h after induction instead seemed to increase toxicity without reaching significance.

Transcription from the strong TetO promotor (doxycycline-inducible promotor) swamps any minor decrease in cellular levels of αSyn. To circumvent this, we devised a protocol with 24 h of doxycycline induction (and SB203580 treatment) in PC12-αSyn/p25α cells followed by doxycycline washout to follow the fate of the induced cohort of αSyn over the following 4 days in culture. Figure 1*C* shows that SB203580 shortened the half-life of αSyn and decreased the cellular pool of αSyn.

We next examined the protein levels and the activation of p38MAPK (by using phosphorylation of Thr180/Tyr182 as a proxy) following SB203580 treatment in PC12 cells expressing αSyn with or without p25α expression. When we compared activation of p38MAPK in PC12 cells expressing αSyn or αSyn/p25α, we observed that total p38MAPK levels were reduced in cells expressing p25α, likely because of enhanced degradation (by next-generation sequencing, there was no altered transcription of p38MAPK isoforms; data not shown) (Fig. 1*D* and *E*). Nevertheless, the level of phosphorylated p38MAPK was significantly increased, indicating a robust activation of an overall decreased pool of p38MAPK by p25α expression (Fig. 1*D* and *F*). Addition of SB203580 to PC12-αSyn/p25α cells entirely and significantly reduced the level of activated p38MAPK to levels seen in control PC12-αSyn cells. In contrast, SB203580 at these low concentrations did not modulate the overall specific activity of p38MAPK in PC12-αSyn cells. We therefore believe that the correction of specific p38MAPK activity (p-p38MAPK/total MAPK) afforded by SB203580 in PC12-αSyn/p25α cells is due to both an inhibition of degradation of p38MAPK and an inhibition of kinase activity (Fig. 1*D*).

### p38MAPK inhibition increases αSyn in lysosomes and modulates lysosomal function

**Figure 2:**
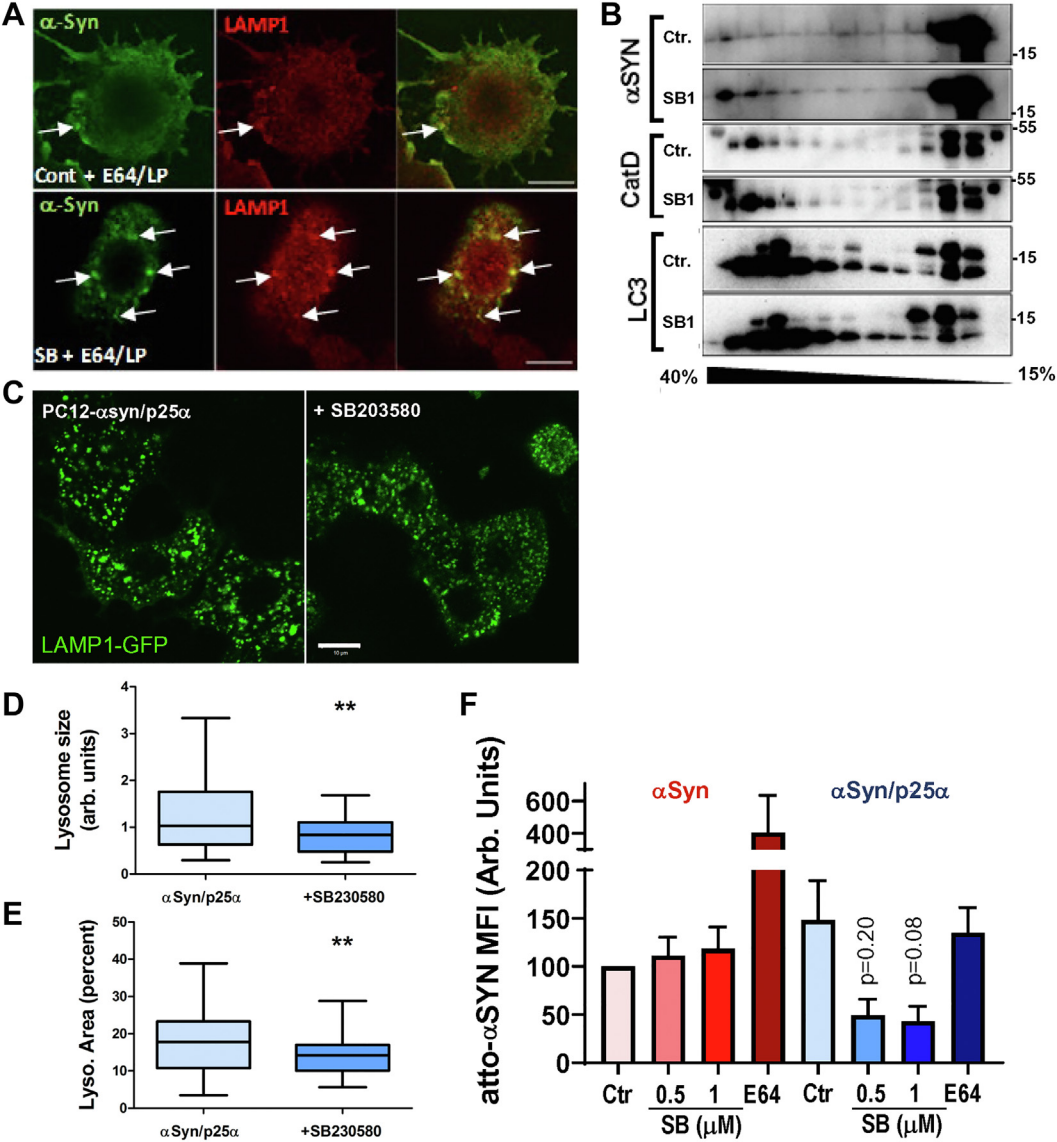
**SB203580 increases lysosomal localization and degradation of αSyn and modulates lysosomal distribution and function.***A*, NGF-differentiated PC12-αSyn/p25α cells were treated with 1 μM SB203580 and protease inhibitors E64 (10 μM) and LP (50/67 μg/ml, respectively) for 2 days before indirect immunofluorescence to reveal αSyn (LB509 mAb) and LAMP-1. Note increased localization of αSyn in LAMP1-positive compartments in SB203580-treated cells (*arrows*). The images are representative of two independent experiments. Bar represents 10 μm. *B*, NGF-differentiated PC12-αSyn/p25α cells were cultured 2 days with or without 1 μM SB203580 before homogenization and fractionation of equal protein loads by sucrose gradient centrifugation. Collected fractions were analyzed by Western blotting for αSyn, cathepsin D, and LC3. Note the increased distribution of αSyn and cathepsin D in heavy lysosomal fractions in SB203580-treated cells. The bands in the LC3 blot represent LC3-I (*upper band*) and LC3-II (*lower band*). The shown blots are representative of two independent experiments. *C*, NGF-differentiated PC12-αSyn/p25α cells stably expressing LAMP1-GFP were treated for 2 days with 1 μM SB203580 before fixation and indirect immunofluorescence with rabbit anti-LAMP1 antibody followed by Alexa488-conjugated goat anti-rabbit antibodies. Bars represent 10 μm. *D* and *E*, images obtained as aforementioned were analyzed by ImageJ to illustrate that SB203580 treatment decreases (*D*) lysosome size and (*E*) fractional area occupied by lysosomes per cell profile. The shown data are derived from 40 cells for each condition (nonparametric Mann–Whitney test, a single experiment of three independent trials with similar significant outcome). *F*, NGF-differentiated PC12-αSyn and PC12-αSyn/p25α cells treated with/without SB203580 for 2 days were incubated with atto-labeled recombinant αSyn for 4 h with/without E64, before cell detachment and immediate flow cytometric analysis (Kruskal–Wallis test, N = 4). All graphs show mean ± SEM, except in *D* and *E*, where mean ± minimum/maximum is depicted. αSyn, α-synuclein; LAMP, lysosome-associated membrane protein; LP, leupeptin/pepstatin; mAb, monoclonal antibody; NGF, nerve growth factor.

Since p38MAPK inhibition decreased αSyn secretion, we examined whether this correlated with an increased transport of αSyn to lysosomes to presumably increase degradation. By qualitative indirect immunofluorescence, SB203580 increased lysosomal localization of αSyn in PC12-αSyn/p25α cells cotreated with leupeptin/pepstatin (LP) and E64 to inhibit lysosomal hydrolysis (Fig. 2*A*). We corroborated this finding by performing sucrose gradient fractionation of PC12-αSyn/p25α cell homogenates (Fig. 2*B*). In the presence of SB203580, an increased pool of αSyn is observed in the heavy lysosomal fractions of the gradient indicating a higher lysosomal transport/import of αSyn. Note that the levels of cathepsin D in heavy fractions were also increased by SB203580, reflecting an enhanced biosynthesis and/or altered trafficking of this protease. Cathepsins D and L are particularly important for αSyn degradation (33, 34), and cell lysates prepared from PC12-αSyn or PC12-αSyn/p25α cells confirmed that SB203580 (albeit nonsignificantly) increases the cellular levels of cathepsin D (Fig. S1, *A* and *B*).

Lysosomal pH was also affected by SB203580. Expression of αSyn in itself seemed to increase lysosomal pH as gauged by the pH-sensitive Lysosensor probe, and this increase was further accentuated by SB203580 independently of p25α expression, though not statistically significant (Fig. S1, *C* and *D*).

SB203580 also markedly changed the subcellular distribution and size of lysosomes. In p25α-expressing PC12 cells, lysosomes tend to cluster and enlarge, an effect counteracted by SB203580, which decreased the abnormally large size of lysosomes and their aggregation in PC12-αSyn/p25α cells, and the fractional cytosolic area occupied by lysosomes (Fig. 2, *C*–*E*) without decreasing their numbers significantly.

To directly assess the effect of these lysosomal changes on αSyn breakdown, we examined the effect of SB203580 on the endolysosomal degradation of fluorophore (atto)-conjugated recombinant αSyn (Fig. 2*F*). Expectedly, in PC12-αSyn/p25α cells, degradation of atto-αSyn was slightly hampered by the expression of p25α (17); however, addition of SB203580 increased αSyn degradation (although without reaching significance, *p* = 0.08). In contrast, in cells expressing only αSyn, SB203580 did not alter αSyn degradation. Addition of 10 μM E64 (cysteine protease inhibitor) to quench lysosomal proteases served as control.

### The effect of p38MAPK inhibition is independent of autophagy inhibition by autophagy-related 5 knockdown or 3-methyladenine

**Figure 3:**
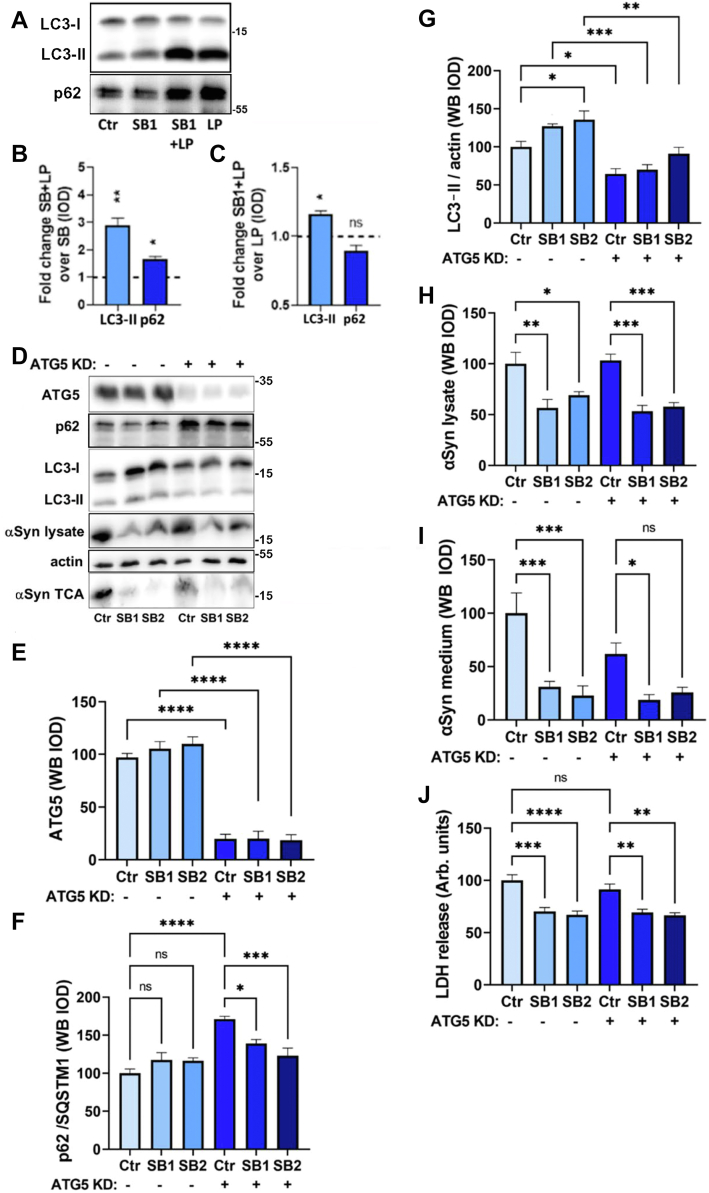
**The decreased secretion and increased degradation of αSyn afforded by SB203580 treatment does not rely on macroautophagy.***A*–*C*, differentiated PC12-αSyn/p25α cells were treated with 1 μM SB203580 with or without leupeptin/pepstatin (LP) for 2 days before lysis and Western blot of LC3 and p62. Graphs show effect of (*B*) LP and (*C*) SB203580 addition, respectively, on LC3-II and p62 protein levels (one-sample *t* test, N = 4). *D*–*I*, NGF-differentiated PC12-αSyn/p25α cells were transduced with lentivectors expressing ATG5 shRNA and then treated with doxycycline with/without SB203580 for 24 h followed by a 2 day chase (minus doxycycline) before Western blot analysis of (*E*) ATG5, (*F*) p62/SQSTM1, (*G*) LC3-II, and (*H*) αSyn and actin in lysates, and (*I*) αSyn and (*J*) LDH levels in conditioned medium. All graphs show mean ± SEM and were analyzed by ordinary one-way ANOVA (N = 4–5). αSyn, α-synuclein; ATG5, autophagy-related 5; LDH, lactate dehydrogenase; NGF, nerve growth factor.

p38MAPK kinases are known to negatively regulate the macroautophagosomal pathway, whereas more specialized forms of autophagy seem to be positively regulated (35, 36, 37). In PC12 cells, we have previously shown that macroautophagy is a predominant pathway of αSyn degradation under basal conditions, and that αSyn secretion is blocked by macroautophagy inhibition (17). For this reason, we examined the disposition of LC3-II and p62 as markers of autophagosomal activity following SB203580 treatment with or without concurrent block of lysosomal proteases with LP inhibitors to asses lysosomal accumulation of LC3-II and p62 and thereby processivity of the autophagosomal pathway. As shown in Figure 3, *A* and *B*, LP treatment alone caused a significant accumulation of both LC3-II and p62. SB203580 treatment in itself caused a significant increase in LC3-II levels, whereas p62 levels remained unaltered (Fig. 3*C*). At higher concentrations of SB203580, the increase in LC3-II levels was even more prominent (Fig. S2*A*). We therefore specifically explored the effect of SB203580 on αSyn turnover under conditions of macroautophagy blockade by transduction with autophagy-related 5 (ATG5) shRNA. To be able to pick up modest changes in αSyn levels in the lysate fraction, a doxycycline pulse-chase protocol (as in Fig. 1*C*) was implemented. As shown in Figure 3, *D*–*G*, ATG5 knockdown reduced protein levels to ca. 20% in PC12-αSyn/p25α cells, and this caused significantly increased levels of p62 and decreased levels of LC3-II, respectively, as would be expected of a macroautophagosomal block. However, despite ATG5 knockdown, SB203580 still decreased secreted levels of αSyn significantly in both lysate and medium fraction (Fig. 3, *H* and *I*). As expected (17), levels of secreted αSyn also trended toward being lowered by ATG5 knockdown in itself (Fig. 3*I*). Importantly, SB203580 lowered cellular toxicity as measured by LDH release also in ATG5 knockdown cells (Fig. 3*J*). These data are supported by experiments with 3-methyladenine (3-MA) inhibition of autophagy, which more dramatically than ATG5 knockdown decreases αSyn secretion (17) in PC12-αSyn/p25α cells (Fig. S2, *B*–*F*). While there is a tendency of SB203580 to lower the cellular content of αSyn across different concentrations of 3-MA, there was no further depression by SB203580 of the dramatic block in αSyn secretion afforded by 3-MA incubation alone (17). These findings were corroborated by qualitative indirect immunofluorescence. In the basal state, αSyn in PC12-αSyn/p25α cells could be localized to LC3-positive autophagosomes, some of which also stained for LAMP1 (Fig. S2*G*). However, in SB203580-treated cells, the colocalization of αSyn with LC3 decreased, alongside an increased colocalization of αSyn and LAMP1. Our results indicate that while p38MAPK inhibition increases autophagy, the SB203580 effect on αSyn turnover is independent of macroautophagy.

Finally, we also addressed proteasomal degradation of αSyn (Fig. S3), which is known to contribute to αSyn degradation (26, 38, 39). Proteasomal blockade by the commonly used inhibitor MG132 (proteasomal inhibition verified by Western blotting of ubiquitin) in itself caused a significant decrease of αSyn secretion (we speculate that a positive regulator of exophagy is a client of the proteasome), but did not influence the significantly more robust SB203580 effect (Fig. S3*A*). MG132 effected an accumulation of αSyn in the lysate fraction, and this was counteracted, although not significantly, by SB203580 (Fig. S3*B*). Overall, the results indicate that the proteasome contributes to degradation of αSyn under basal conditions but is not likely to be involved in the effects of SB203580 on αSyn turnover and secretion.

### Pharmacological blockade of lysosome fusion to mimic p25α effect does not confer SB203580 sensitivity to PC12-αSyn cells

**Figure 4:**
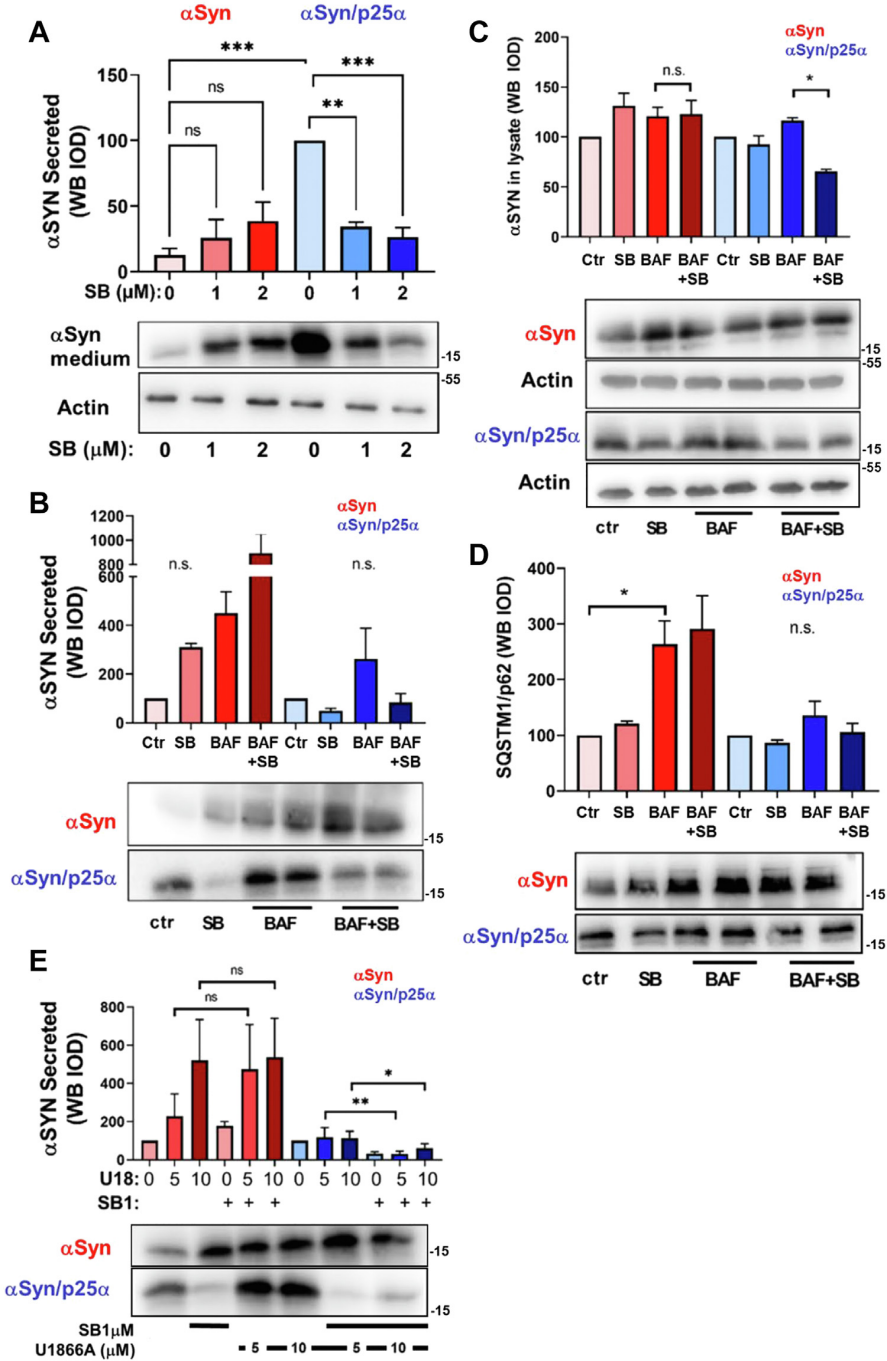
**Pharmacological block of lysosome fusion in PC12-αSyn cells does not confer susceptibility to SB203580.***A*, NGF-differentiated PC12 expressing αSyn alone or together with p25α were treated with 1 or 2 μM SB203580 for 2 days before analysis of secreted αSyn and actin in the lysate (Kruskal–Wallis test, N = 3 for αSyn-expressing cells and N = 8–10 for cells expressing both αSyn and p25α). *B*, NGF-differentiated PC12 cells expressing either αSyn alone or together with p25α were treated with doxycycline and SB203580 for 24 h and then chased in the absence of doxycycline with/without SB203580 or bafilomycin A1 as indicated for a further 2 days before analysis of (*B*) secreted αSyn or (*C*) lysate levels of αSyn or (*D*) p62/SQSTM1 (Kruskal–Wallis test, N = 3). *E*, NGF-differentiated PC12 cells expressing either αSyn alone or together with p25α were incubated with U18666A at the indicated concentrations for 48 h in the presence of 1 μM SB203580 and then conditioned medium was analyzed for αSyn content (N = 4–5). All graphs show mean ± SEM and were analyzed by the Kruskal–Wallis test. αSyn, α-synuclein; NGF, nerve growth factor.

To expand our observations, we also examined the effect of p38MAPK inhibition in PC12 cells only expressing αSyn. Surprisingly, SB203580 treatment did not ameliorate αSyn secretion from cells without p25α expression but rather enhanced release, albeit not significantly (Fig. 4*A*). This correlates with the lack of effect of SB203580 on the specific activity of p38MAPK in PC12-αSyn cells (Fig. 1*D*). A cardinal effect of p25α in the PC12 cell model is the impediment of autophagosome and lysosome fusion (17), which is sufficient to mediate activation of the JNK2 stress kinase (32). We therefore speculated that the pronounced activation of p38MAPK in PC12-αSyn/p25α cells could also be due to lysosomal stress. If so, it should be possible to confer SB203580 susceptibility to PC12 cells expressing only αSyn by treatment with drugs that similarly to p25α block or impede lysosomal fusion. We therefore studied the effects of bafilomycin A1 or U18666A on SB203580-modulated αSyn metabolism in PC12 cells expressing αSyn with or without p25α. Bafilomycin A1, which blocks late endosome/autophagosome fusion with lysosomes, provokes a greatly increased release of αSyn from PC12-αSyn cells (Fig. 4*B*) because accumulating autophagosomes are exocytosed (17). In contrast, in PC12-αSyn/p25α cells, bafilomycin only moderately increases αSyn release because its effect to inhibit lysosomal fusion is additive to that already established by p25α expression alone. In absolute numbers, the release of αSyn in the basal state was increased 7.9 ± 0.8-fold in PC12-αSyn/p25α cells compared with PC12-αSyn cells (Fig. 4*A*). SB203580 treatment lowered (nonsignificantly) the bafilomycin A1-induced αSyn release in PC12-αSyn/p25α cells (Fig. 4*B*; 68% reduction) and significantly decreased the accumulation of αSyn in the lysate fraction induced by bafilomycin A1 (Fig. 4*C*). However, in PC12-αSyn cells, SB203580 conversely aggravated the bafilomycin A1 effect on αSyn release and did not decrease cellular levels of αSyn. Lysate levels of p62/SQSTM1 were included as controls of lysosomal fusion block and expectedly increased upon bafilomycin A1 treatment in PC12-αSyn cells, less so in PC12-αSyn/p25α cells because of the p25α-mediated fusion block (Fig. 4*D*). U18666A is a cationic amphiphilic drug known to perturb late endosomal dynamics and lysosomal fusion by hampering cholesterol transport out of late endosomes (40). U18666A caused a several-fold increase in αSyn secretion from PC12-αSyn cells, and SB203580 treatment did not affect this release (Fig. 4*E*). In contrast, in PC12-αSyn/p25α cells, U18666A did not alter αSyn secretion levels significantly, yet did not interfere with the ability of SB203580 to significantly reduce αSyn secretion as observed previously for bafilomycin A1.

In conclusion, endolysosomal aberration does not confer p38MAPK activation and susceptibility to SB203580 in PC12-αSyn cells, and we surmise that the strong activation of p38MAPK elicited by p25α is due to other effects than lysosomal fusion impediment.

### shRNA knockdown of the p38MAPK α-isoform replicates the effects of SB203580

**Figure 5:**
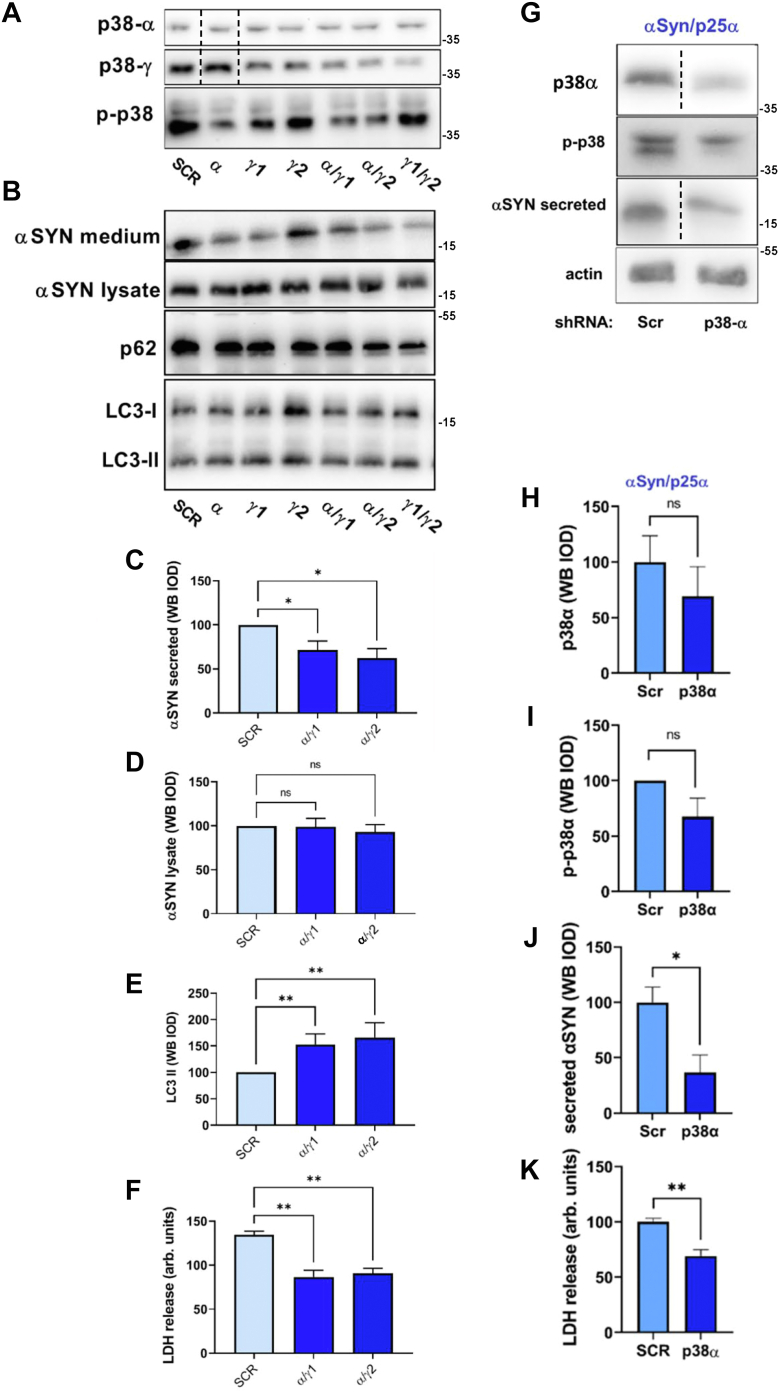
**shRNA knockdown of p38MAPK-α reproduces the effects of SB203580 treatment.***A*, differentiated PC12-αSyn/p25α cells were transduced with lentivectors expressing shRNA to p38MAPK-α (one shRNA) and p38MAPK-γ isoforms (two shRNAs), incubated with doxycycline for 2 days, and subsequently lysed and Western blotted for expression of p38MAPK isoforms (using anti-p38MAPKα antibody 9212 from Cell Signaling) or phosphorylated p-p38MAPK. Cropped lanes are derived from the same membrane. *B*, the same cells were analyzed by Western blotting for expression of αSyn in the conditioned medium, and αSyn, LC3, and p62 in the lysate. *C*–*E*, quantitation of Western blot band absorbances from aforementioned experiment for double p38MAPK-α and p38MAPK-γ knockdown for (*C*) αSyn in conditioned medium, or (*D*) αSyn, and (*E*) LC3-II in the lysate fraction. (Kruskal–Wallis test, N = 4–6). *F*, LDH release from the same cells (ordinary one-way ANOVA, N = 4). *G*, differentiated PC12-αSyn/p25α cells were transduced with lentivectors expressing shRNA to p38MAPK-α and then analyzed as aforementioned except anti-p38MAPK-α antibody (StressMarq Biosciences; SMC-152D) was used. Cropped lanes are derived from the same membrane. *H* and *I*, the graphs show quantitation of levels of p38MAPK-α isoform (unpaired *t* test, N = 4) and active (phosphorylated) p38MAPK (one-sample *t* test, N = 4). *J*, secretion of αSyn to the medium (unpaired *t* test, N = 3) as performed in (*G*). *K*, LDH release to the medium (unpaired *t* test, N = 4). All graphs show mean ± SEM. αSyn, α-synuclein; LDH, lactate dehydrogenase.

It seems clear that SB203580 has effects on autophagy that are not directly related to p38MAPK inhibition (37, 41). To affirm direct involvement of p38MAPK in the effects observed upon SB203580 treatment, we performed a series of experiments with knockdown of p38MAPK isoforms. We first analyzed differentiated PC12 neurons for expression of p38MAPK isoforms (Fig. S4). PC12 cells expressed only p38MAPK-α and p38MAPK-γ isoforms, and consequently, we therefore partially (near full knockdown was toxic to differentiated cells) knocked down p38MAPK-α or p38MAPK-γ alone or in combination in PC12-αSyn/p25α cells by lentiviral shRNA transduction (Fig. 5*A*).

We observed that PC12-αSyn/p25α cells transduced specifically with the α-isoform shRNA alone, or in combination with γ-isoform shRNA, demonstrated decreased levels of p-p38MAPK (Fig. 5*A*). This correlated with a significantly decreased αSyn secretion, increased LC3-II levels, and lowered αSyn/p25α-mediated toxicity (Fig. 5, *B*, *C*, *E* and *F*) without altering cellular levels of αSyn significantly (Fig. 5*D*). Analysis of p38MAPK-α in this series of experiments was unfortunately performed with an antibody (Cell Signaling; catalog no.: 9218P; Fig. 5*A*) that did not convincingly demonstrate knockdown of the α-isoform (despite observed effects). Based on our results and the known isoform-selective inhibitory activity of SB203580 (42), we speculated that knockdown of the α-isoform alone would be sufficient to replicate the SB203580 observations. We therefore repeated α-isoform knockdown experiments this time using polyclonal antibody SMC-152D from StressMarq Biosciences for analysis (Fig. 5*G*). This antibody confirmed that the α-isoform is reduced by knockdown, correlating with decreased levels of phosphorylated (active) p38MAPK (Fig. 5, *G*–*I*), and a significantly decreased level of secreted αSyn (Fig. 5*J*), as well as LDH level (Fig. 5*K*). We conclude that the SB203580 effects observed can specifically be replicated by knockdown of p38MAPK α-isoform.

### p38MAPK inhibition effect on αSyn turnover depends neither on LAMP2a (CMA) nor on Ndfip1–Nedd4 mediated import into endolysosomes

As we observe an increased localization and degradation of αSyn in late endolysosomal compartments independently of macroautophagy, we wondered whether endolysosomal import of αSyn could be direct through either LAMP2a or Ndfip1, which operate through two different mechanisms to selectively feed cytosolic proteins into lysosomes and late endosomes, respectively.

**Figure 6:**
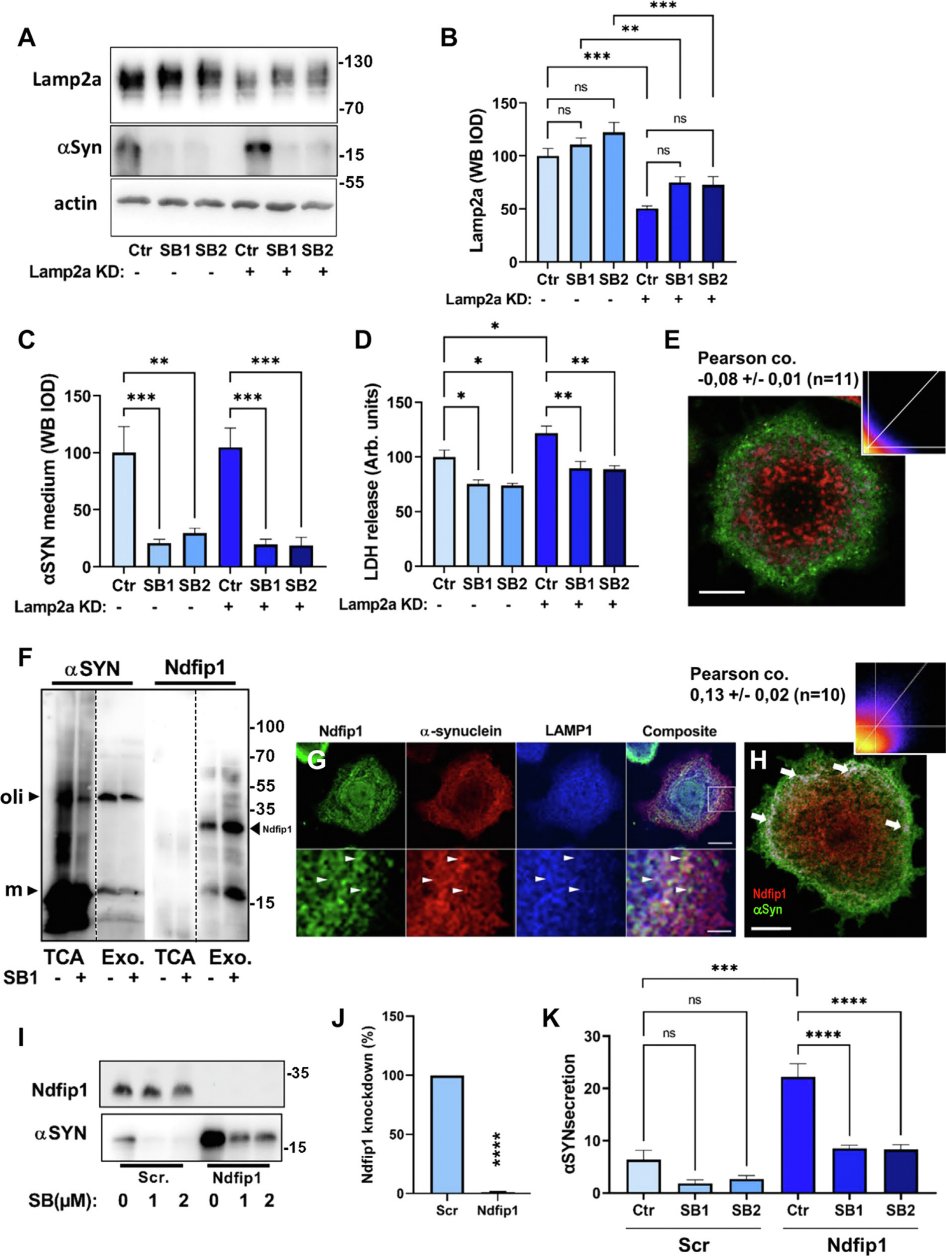
**Neither chaperone-mediated autophagy nor Nedd4/Ndfip-1 accounts for the SB203580 effect to increase αSyn turnover.***A*–*D*, NGF-differentiated PC12-αSyn/p25α cells were transduced with lentivectors expressing scrambled or LAMP2a shRNA, and after 2 days, lysates were analyzed for LAMP2a and actin, and the medium for secreted αSyn and LDH. *B*, quantitation of LAMP2a shRNA knockdown effect (one-way ANOVA, N = 5). *C*, conditioned medium from PC12-αSyn/p25α cells expressing scrambled or LAMP2a shRNA and treated with/without SB203580 was analyzed for αSyn by Western blotting (one-way ANOVA, N = 5). *D*, LDH content in conditioned medium (one-way ANOVA, N = 5). *E*, indirect immunofluorescence of αSyn (mAb LB509; in *green*) and LAMP2a (in *red*) in PC12-αSyn/p25α cells treated with 1 μM SB203580 and 10 μM E64 to inhibit lysosomal proteases. A representative image after analysis in ImageJ with a colocalization algorithm is shown, where colocalized pixels (if present) appear *white*. Note the peripheral localization of αSyn-positive vesicles, mostly separated from the perinuclear LAMP2a-positive lysosome pool (*inset*; pixel intensity scatter plot) yielding a Pearson coefficient of −0.08 ± 0.01 (n = 11 cells from two experiments). Bar represents 10 μm. *F*, conditioned medium from PC12-αSyn/p25α cells treated with/without SB203580 was either differentially centrifuged to obtain a washed exosomal pellet (Exo) or TCA-precipitated (TCA), and fractions were then analyzed by Western blotting for αSyn and Ndfip1. Note the presence of αSyn monomer (m) and oligomers (oli) in both exosome and concentrated supernatant (TCA) fractions, and that SB203580 increases Ndfip1 in exosomes, while decreasing αSyn. The shown blot is representative of two independent trials; all lanes are derived from the same membrane for αSyn and Ndfip1, respectively. Molecular weight markers are indicated. *G*, indirect immunofluorescence to show αSyn (mAb LB509) in relation to Ndfip1 and LAMP1 in PC12 cells expressing αSyn/p25α under basal conditions. *Arrows* indicate colocalization of αSyn, Ndfip1, and LAMP1. The shown images are representative of two independent experiments. The *squared area* in *top right panel* is depicted at higher magnification in the *lower row of panels*. *Bar upper panels* represent 10 μm; *bar lower panels* represent 2.5 μm. *H*, a representative image after analysis in the ImageJ colocalization algorithm is shown, where αSyn (*red*) and Ndfip1 (*green*) pixels appear *white* when colocalized (*arrows*). Analysis of αSyn/Ndfip1 colocalization yielded a Pearson coefficient of 0.13 ± 0.02 (n = 10 cells from a single representative experiment of two). Bar represents 10 μm. *I*–*K*, NGF-differentiated PC12-αSyn/p25α cells were transduced with lentivectors expressing scrambled or Ndfip1 shRNA and then treated with doxycycline for 2 days with/without SB203580 as indicated. *I*, representative Western blots are shown for Ndfip1 protein in the lysate and for secreted αSyn in the conditioned medium. *J*, quantitation of Ndfip1 knockdown (one-sample *t* test, N = 4). *K*, quantitation of αSyn release from PC12-αSyn/p25α cells that received scrambled or Ndfip1-shRNA treated or not with 1 or 2 μM SB203580 as indicated (one-way ANOVA, N = 4). All graphs show mean ± SEM. αSyn, α-synuclein; LAMP, lysosome-associated membrane protein; LDH, lactate dehydrogenase; mAb, monoclonal antibody; NGF, nerve growth factor; TCA, trichloroacetic acid.

αSyn contains a peptide (^95^DDAVK) that conforms to the consensus motif for Hsc70-mediated binding and internalization of cargo proteins into the lysosome lumen by way of the membrane protein LAMP2a. This process is called CMA (28, 43), and to block it, we knocked down LAMP2a by lentiviral transduction to achieve a significant ∼50% reduction of protein in PC12-αSyn/p25α cells (Fig. 6, *A* and *B*). We consistently observed that SB203580 treatment increased the molecular weight of LAMP2a slightly (Fig. 6*A*) and a nonsignificant trend toward increased levels of LAMP2a (Fig. 6*B*). However, knockdown of LAMP2a did not in itself cause an altered secretion of αSyn, indicating that CMA is a lesser degradation pathway for αSyn under basal conditions, and importantly, knockdown did not interfere with the ability of SB203580 to decrease levels of secreted αSyn in PC12-p25α/αSyn cells (Fig. 6*C*). Neither did LAMP2a shRNA abrogate the decreased secretion of LDH following SB203580 treatment (Fig. 6*D*). These results are corroborated by immunofluorescence studies that showed very little colocalization between peripherally distributed αSyn and more perinuclear LAMP2a-positive compartments (Fig. 6*E*), yielding a slightly negative Pearson coefficient of −0.08 ± 0.01 (N = 11 cells). Collectively, our findings demonstrate that SB203580 effects on αSyn turnover cannot be ascribed to LAMP2a and CMA.

The ubiquitin ligase Nedd4 has been identified as a central hub in αSyn metabolism in different cellular systems (30, 44), but the role of Ndfip1, the late endosomal-resident receptor for Nedd4, has never been addressed directly in the literature. Given the role of Nedd4 in αSyn turnover (45, 46), and the possibility that Ndfip1 could act to mediate αSyn import (as with other substrates) *via* ESCRT and exosomal import into late endosomes, we decided to examine any role of Ndfip1 in the observed effects of SB203580. Nedd4 and Ndfip1 have been identified in exosomes (47), and so has αSyn (17, 48, 49). We therefore isolated exosomes from conditioned medium derived from PC12-αSyn/p25α cells treated with or without 1 μM SB203580 and compared this fraction to aliquots of conditioned medium precipitated with TCA (Fig. 6*F*). The Ndfip1 level in exosomes was increased by SB203580, while being undetectable in the TCA-precipitated fraction. Levels of αSyn in the exosomal pellet were moderately decreased by SB203580, but relative to total levels of αSyn secreted (TCA fraction), the majority being soluble, SB203580 actually increased the proportion of αSyn contained in exosomes.

Next, we performed indirect immunofluorescence of PC12-αSyn/p25α cells to learn that αSyn and Ndfip1 showed a high degree of colocalization, and further, a fraction of these vesicles was also positive for LAMP1 (Fig. 6*G*). By ImageJ colocalization analysis, a Person coefficient of 0.13 ± 0.02 (n = 10) was obtained for colocalization between αSyn and Ndfip1, predominantly in peripheral positions of the cell (Fig. 6*H*).

We therefore proceeded to perform shRNA knockdown of Ndfip1 in PC12-αSyn/p25α cells. Ndfip1 protein levels could be successfully reduced by 99% without cytotoxicity (Fig. 6, *I* and *J*). We first noted that under basal conditions, knockdown of Ndfip1 caused a mean fivefold increase of secreted αSyn levels (Fig. 6*K*). However, the absence of Ndfip1 did not interfere with the ability of SB203580 to suppress secretion of αSyn. Thus, Ndfip1 plays a major role in regulating αSyn uptake into endolysosomes under basal conditions but does not explain the SB203580 effect.

### The ability of SB203580 to reduce αSyn secretion relies on Hsc70 and ESCRT-I subunit TSG101

**Figure 7:**
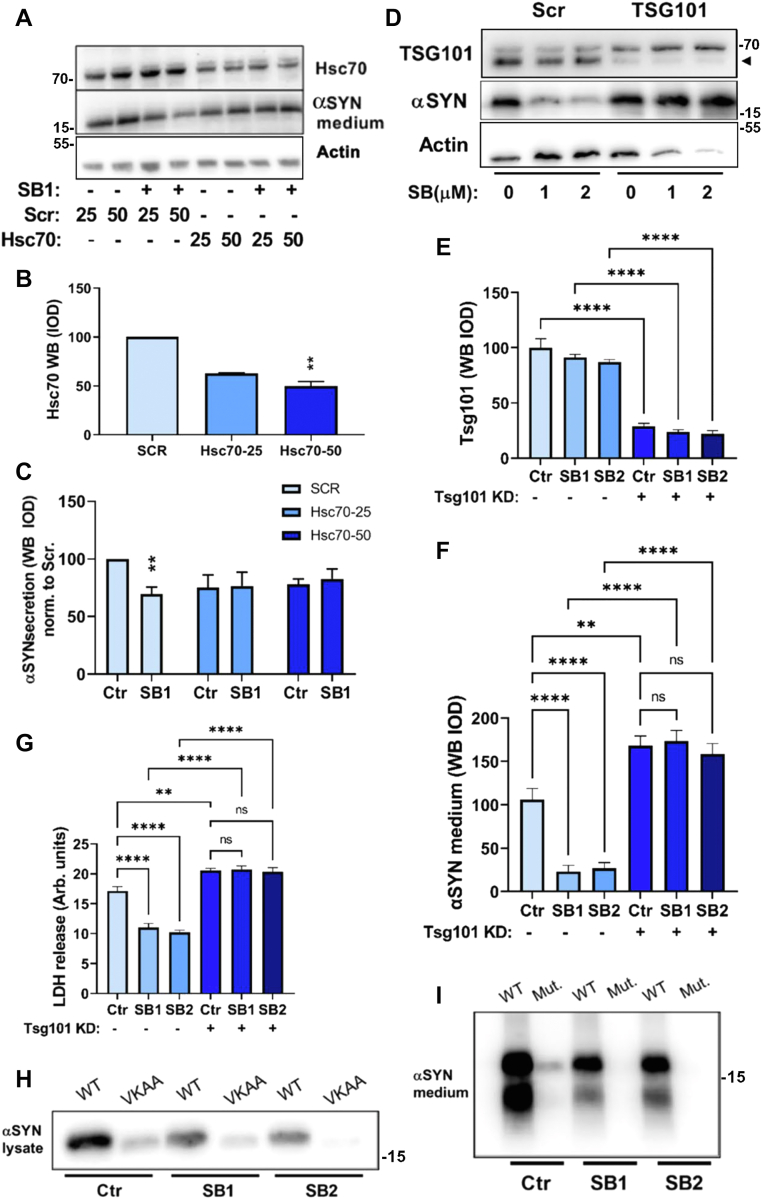
**The SB203580 effect on αSyn release is attenuated by knockdown of either Hsc70 or ESCRT-component TSG101.***A*–*C*, NGF-differentiated PC12-αSyn/p25α cells were transduced with doses of either 25 or 50 μl of lentivector expressing scrambled or Hsc70 shRNA and then induced with doxycycline to express αSyn and p25α for further 2 days with/without SB203580 treatment as indicated. Lysate and conditioned medium were then collected for Western blot analysis of Hsc70 knockdown relative to actin and αSyn secretion, respectively. *B*, quantitation of Hsc70 knockdown under basal conditions by Western blot (one-sample *t* test, N = 4), and (*C*) secreted αSyn with or without SB203580 (Kruskal–Wallis test, N = 6). In *B* and *C*, cells received scrambled virus in a dose of 25 μl. *D*–*G*, NGF-differentiated PC12-αSyn/p25α cells were transduced with lentivectors expressing scrambled or TSG101 shRNA and after 2 days of αSyn/p25α expression with/without SB203580 treatment as indicated. *D*, cells and conditioned medium were collected for Western blot analysis of TSG101 knockdown and actin, and αSyn secretion, respectively. The *arrowhead* marks the position of TSG101. The upper band did not appear consistently. *E*, quantitation of TSG101 knockdown (one-way ANOVA, N = 4) and (*F*) αSyn release (one-way ANOVA, N = 4). *G*, conditioned medium from the aforementioned experiment was analyzed for LDH release (one-way ANOVA, N = 4). All graphs show mean ± SEM. *H*, lysate or (*I*) conditioned medium from PC12-αSyn/p25α cells expressing either αSyn wildtype or ^95^VKKAA mutant, and treated or not with 1 or 2 μm SB203580 as aforementioned, was analyzed by Western blotting for αSyn levels. The blots shown are representative of two independent experiments. Note that anti-αSyn mAb from BD (and two other anti-αSyn antibodies investigated) used for the purpose recognizes the mutated αSyn with low efficiency. αSyn, α-synuclein; ESCRT, endosomal sorting complex required for transport; LDH, lactate dehydrogenase; mAb, monoclonal antibody; NGF, nerve growth factor.

By the process of microautophagy, cytosolic cargo is introduced into the lumen of late endosomes by invagination and budding of the limiting membrane to produce intraluminal vesicles (ILVs), in a mechanism that requires the ESCRT complex (24, 50). In “classical” microautophagy, there is a requirement for Hsc70 to engage with substrate and bring it to the endolysosomal membrane (29). While Hsc70 levels were not altered by p25α expression or SB203580 treatment (data not shown), we decided to knock down the protein to analyze effects on αSyn turnover. Protein levels of Hsc70 could be reduced to ca. 50% (without toxicity) (Fig. 7, *A* and *B*), and concurrently, we observed that SB203580 lost its effect to lower secretion of αSyn (Fig. 7, *A* and *C*). We expanded this observation by knocking down TSG101, an ESCRT-I protein required for microautophagy (29). Knockdown of TSG101 (Fig. 7, *D* and *E*) caused not only a 1.5-fold increased secretion of αSyn compared with control cells but also entirely abolished the effect of SB203580 to decrease αSyn secretion (Fig. 7, *D* and *F*). This also correlated with a loss of SB203580 to ameliorate cytotoxicity of αSyn and p25α expression (Fig. 7*G*).

The role played by Hsc70 in microautophagy is cargo recruitment, which depends on the Hsc70 binding consensus motif in substrates, in αSyn, the ^95^VKKDQ sequence (25). Accordingly, we mutated this motif to ^95^VKKAA (25) and compared the turnover of αSyn wildtype and mutant protein in relation to SB203580 treatment in a doxycycline pulse-chase protocol. Of note, mutated αSyn had a slightly higher electrophoretic mobility than wildtype (25) and was detected on the membrane with lower efficiency by several anti-αSyn antibodies tested. By indirect immunofluorescence of paraformaldehyde (PFA)-fixed cells, however, there seemed to be no impediment of antibody binding, and both wildtype and mutated αSyn cell populations expressed comparable amounts of αSyn. Surprisingly, mutation of the Hsc70 interaction motif did not interfere with the capacity of SB203580 to lower lysate or secreted levels of αSyn in PC12-αSyn/p25α cells (Fig. 7, *H* and *I*). In conclusion, the requirement of TSG101 for the SB203580 effect is not directly tied to classical microautophagy but rather depends on an intact ESCRT system.

### SB203580 also mitigates provoked α-synucleinopathy in a primary neuron model of αSyn fibril seeding

To test the effects of SB203580 in a translational model, we established an αSyn seeding model using primary cortical neurons from mice overexpressing human wildtype αSyn (51). Serine-129 of αSyn is highly phosphorylated in synucleinopathic lesions of post mortem PD and Lewy body disease brains (4) and is often used as an indicator of disease progression. The high-content imaging scanner Cellomics was used to quantify the p-Ser129 signal after indirect immunofluorescence staining. Human αSyn fibrils were sonicated to produce small fragments with increased seeding potency (52) prior to neuronal treatment (Fig. S5, *A*–*C*). Addition of human preformed eukaryotic fibrils to cortical neurons induced a 113-fold signal increase in p-Ser129 staining intensity relative to untreated control neurons (Fig. S5, *D*–*F*). To verify that the p-Ser129 signal measured was indeed dependent on the engagement of endogenous (transgenic) human αSyn by the seed fibrils, we silenced endogenous αSyn by means of siRNA (siSNCA), which reduced the signal by 96% compared with siCtr (Fig. S5, *D*–*F*). As observed previously (53), the neurons showed nuclear p-Ser129 staining independently of the addition of αSyn fibrils; therefore, the nuclear staining was excluded from analysis by the Cellomics algorithm. In addition, we used two different Cisbio Homogenous Time-Resolved Fluorescence assays to show that the level of αSyn p-Ser129 correlates with αSyn aggregation in a fibril concentration-dependent manner (Fig. S5*G*).

**Figure 8:**
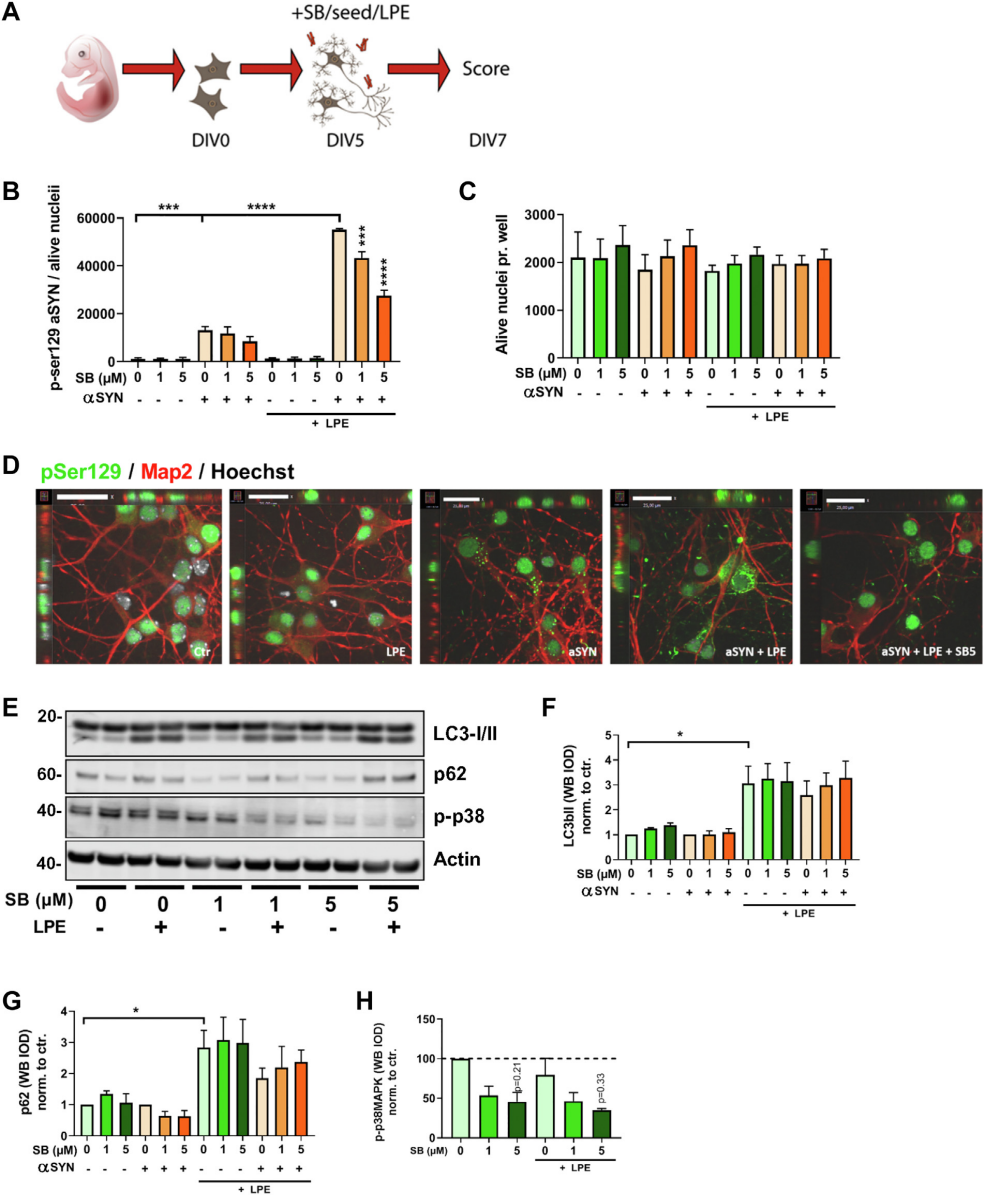
**SB203580 decreases p-ser129 αSyn staining in primary neurons.***A*, primary mouse cortical neurons expressing transgene human αSyn were treated with 1 μg/ml αSyn fibrils ±1 or 5 μM SB203580 at DIV5 in the further presence or not of lysosomal inhibitors leupeptin, pepstatin, and E64 (LPE) and harvested DIV7. *B* and *C*, neuronal cultures were fixed and stained with primary antibodies for (*B*) p-Ser129 αSyn and (*C*) Hoechst (to assess live nuclei) for quantitation by Cellomics Array Scanner analysis (ordinary one-way ANOVA, N = 3). *D*, representative spinning disc confocal microscopy images of neuronal cultures stained for p-Ser129 (*green*), Map2 (*red*), and Hoechst (*gray*) from a Cellomics experiment as aforementioned; bars represent 32 μm. *E*, cell lysates prepared from DIV7 neuronal cultures, treated with SB203580 and/or LPE in the absence of αSyn fibrils as indicated, were Western blotted with anti-LC3, anti-p62, anti-p-p38MAPK, or anti-actin antibodies as shown. *F*–*H*, quantitation of aforementioned immunoblots was performed for (*F*) LC3-II, (*G*) p62/SQSTM1, and (*H*) p-p38MAPK (without αSyn fibrils) (ordinary one-way ANOVA for *F* and *G* and Kruskal–Wallis test for *H*, N = 3). All graphs show mean ± SEM. αSyn, α-synuclein; DIV, days *in vitro*.

Next, we sought to determine the effects of SB203580 on primary neurons (Fig. 8*A*). Using the Cellomics assay, SB203580 showed a slight reduction of the p-Ser129 signal induced by the addition of fibrillar αSyn seeds (Fig. 8*B*). However, when we also added protease inhibitors (LPE) to mimic lysosomal deficiency, which expectedly increased the p-Ser129 staining, we found that SB203580 significantly and dose-dependently decreased the intensity of p-Ser129 staining (SB1 µM: 21%, 95% confidence interval = [6;36] and SB5 µM: 50%, 95% confidence interval = [17;82]). Using the Cellomics software to score the number of viable cell nuclei, none of the treatments affected cell death within the experimental time frame (Fig. 8*C*). Furthermore, spinning disk confocal microscopy supported the notion that LPE increased the intracellular p-Ser129 staining and that SB203580 reduced this signal (Fig. 8*D*).

We also tested the effect of SB203580 on autophagosomal marker proteins LC3II and p62/SQSTM1 using Western blot analysis (Fig. 8, *E*–*G*). The addition of protease inhibitors more than doubled the LC3II and p62 signals, indicative of an autophagosomal block, which was not overcome by SB203580 treatment. SB203580 seemed to dose-dependently decrease the p-p38MAPK signals by Western blot analysis though not with statistical significance (Fig. 8*H*). In thread with our observations in PC12 cells, SB203580 was only effective early in the pathological seeding process. If SB203580 was administered 5 days after seeding initiation, it was no longer able to reduce the p-Ser129 αSyn signal (Fig. S6).

To summarize, we find that SB203580 decreases the intracellular fibril–induced αSyn p-Ser129 signal in primary neurons, and that this effect is not attributable to upregulation of macroautophagy.

## Discussion

Once initiated, neurodegenerative disease is believed to spread in the brain by the secretion of aggregates of endogenous nerve cell proteins in a proteotoxic form that confers templated misfolding and aggregation disease to healthy neurons after uptake (23, 54). We show here that p38MAPK inhibition *via* a degradative mechanism, that depends on the ESCRT machinery, lessens cytotoxicity and αSyn secretion in two different cellular models of α-synucleinopathy.

Both JNK (32) and p38MAPK (this study) are strongly activated by p25α expression in PC12 cells. The autophagosomal fusion block instilled by p25α (or bafilomycin) activates JNK stress kinase (32), but our attempts to reproduce the pathological effects of p25α expression, including p38MAPK activation and thereby SB203580 susceptibility, in PC12-αSyn cells by conferring experimental autophagosome–lysosome fusion block were unsuccessful. Our rescue experiments in *C. elegans* (A. O., unpublished results) suggest that p38MAPK may be activated as part of an axonal injury program that relies on DLK and p38MAPK signaling in at least *C. elegans* (31). Other p25α-mediated changes, including hyperacetylation (17), of the microtubule network are also known to induce and/or amplify DLK and p38MAPK activity (55, 56).

Currently, several p38MAPKα agonists are in various phases of clinical trials for their use against different ailments (57). In neurodegenerative disease in particular, p38MAPKα agonists dampen neuroinflammation, but recent data indicate that p38MAPK inhibition in neurons themselves have beneficial effects that may be tied to the function of degradative mechanisms (57, 58, 59).

### p38MAPK inhibition opposes αSyn accumulation while decreasing secretion

We used SB203580 at concentrations well below the typical (10 μM or more), limiting potential off-target effects, which are of particular concern in relation to autophagy (41). SB203580 inhibits only p38MAPK-α and p38MAPK-β (42), whereof differentiated PC12 neurons only express p38MAPK-α; therefore we conclude that the predominant effect of SB203580 is on p38MAPK-α, which is corroborated by our p38MAPK-α shRNA knockdown experiments.

The beneficial effect of p38MAPK inhibition in PC12 cells was tied to p25α expression as SB203580 in its absence aggravated αSyn secretion and cell death. We therefore speculate that p25α expression installs a coincident factor or condition (perhaps axonal injury program) required for strong p38MAPK activation and for inhibition to have an effect.

The effect of SB203580 in PC12-αSyn/p25α cells was dependent on concurrent administration with doxycycline used for p25α and αSyn transgene induction. This phenomenon was perhaps also recapitulated in primary neurons where SB203580 only had effect when added early in the seeding process before prominent aggregation sets in. As the ESCRT-dependent mechanism can only accept cargo of limited size (an ILV being 40–60 nm), we find it likely that physical restraints preclude the internalization and degradation of larger αSyn aggregates developing over time in either cellular model.

### Degradation mechanism of αSyn

**Figure 9:**
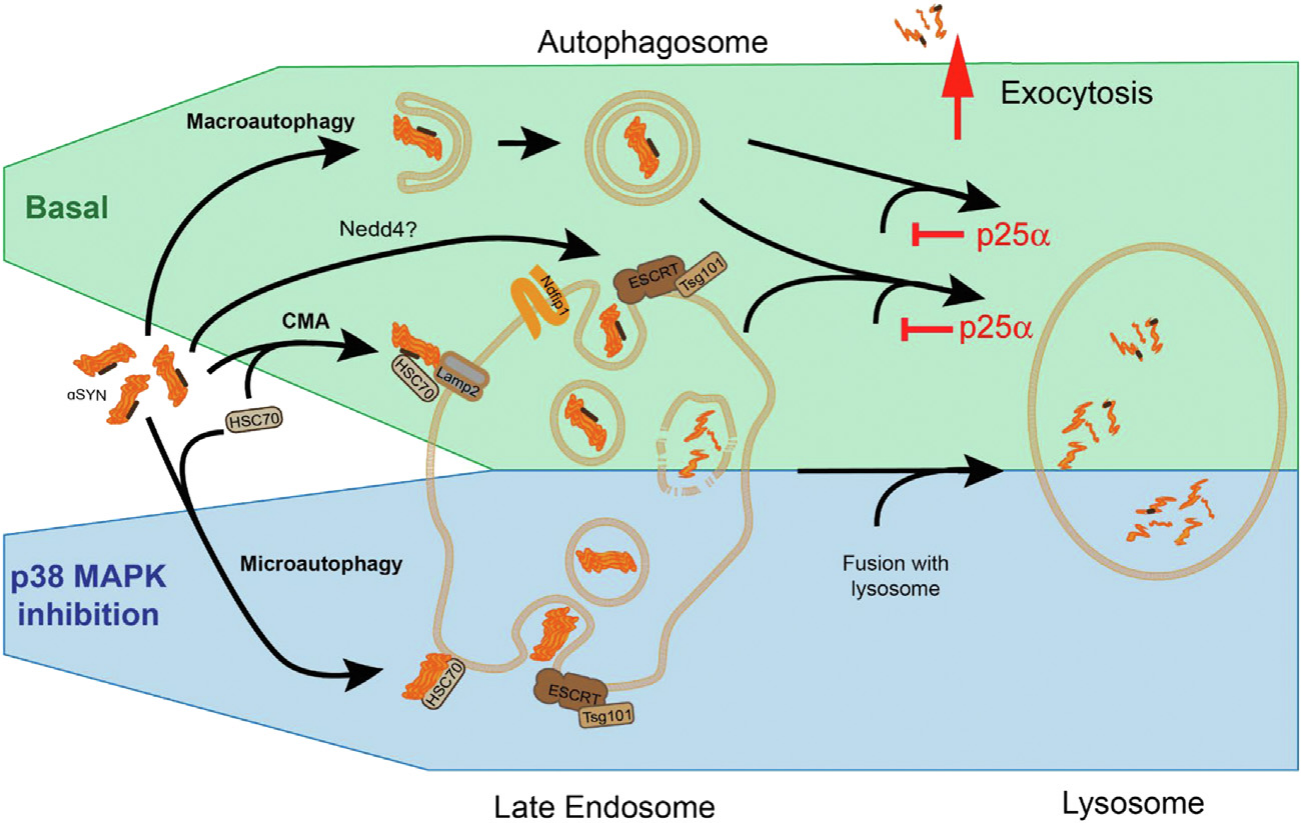
**αSyn degradation under basal conditions and p38MAPK inhibition.** Under basal conditions αSyn is degraded by multiple mechanisms first and foremost by macroautophagy and Ndfip1-mediated internalization of αSyn; however, chaperone-mediated autophagy (LAMP2a and Hsc70) also contributes. When p25α-mediated p38MAPK activation is opposed pharmacologically or genetically, the majority of αSyn is turned over in an ESCRT-dependent process relying on Hsc70 and TSG101, and αSyn degradation commences in late endosomes. Under basal conditions, lysosome fusion with autophagosomes and amphisomes (the fusion product of an autophagosome with a late endosome) is partially blocked by p25α, which results in their exocytosis and release of αSyn. In contrast, under conditions of p38MAPK inhibition, ESCRT-dependent αSyn import and degradation begins in the late endosome, and the endosomal pathway runs to completion to deliver αSyn to lysosomes for degradation. αSyn, α-synuclein; ESCRT, endosomal sorting complex required for transport; LAMP, lysosome-associated membrane protein.

αSyn is known to be degraded by multiple mechanisms, also in PC12 cells, including CMA (26), macroautophagy (17, 26), and the proteasome (38, 39), but the present study constitutes an exhaustive examination of the diverse αSyn degradation pathways under conditions of overexpression in this commonly used cell line. We find that all these mechanisms take part in αSyn degradation under basal conditions, and we further add Ndfip1 as an important mediator of αSyn degradation in PC12 cells. The macroautophagosomal pathway (17) and Ndfip1 clearly has the largest impact on αSyn degradation and secretion, the latter a proxy of αSyn accumulation, under basal conditions (Fig. 9).

The E3 ubiquitin ligase Nedd4 promotes the endolysosomal degradation of αSyn in a process that requires components of the ESCRT complex (30, 44). The transmembrane Ndfip1 protein localizes to late endosomes where it binds and activates several ubiquitin ligases including Nedd4, but Ndfip1 has not been tied directly to αSyn metabolism before. Ndfip1 knockdown increased release of αSyn fivefold, which can be explained by the augmented uptake (more αSyn substrate) into autophagosomes and their exocytosis under conditions of lysosomal fusion block (accumulation of autophagosomes cannot be tolerated (60)). However, the ability of SB203580 to reduce levels of secreted αSyn was not impeded by neither Ndfip1 nor CMA/LAMP2a knockdown, another direct import route into endosomes known to contribute to αSyn degradation (25, 26).

Generally speaking, p38MAPK has been noted to negatively regulate macroautophagy *in vitro* and *in vivo* (35, 59, 61) while it may promote other forms of autophagy (36, 43) (see Refs. (37, 57) for discussion). In agreement with this, we find here that SB203580 or p38MAPKα knockdown consistently increases macroautophagy as measured by increased LC3-II protein levels. Nevertheless, the ability of SB203580 to reduce cytosolic or secreted levels of αSyn was not abrogated by macroautophagy inhibition.

By the process of microautophagy, soluble cytosolic targets containing an Hsc70-binding consensus motif (KERFQ; ca. one-third of the cytosolic proteome including αSyn) are guided to the late endosomal membrane by the lipid-binding propensity of Hsc70. Substrates are then internalized by the core ESCRT machinery into ILVs for degradation in the late endosome or following lysosome fusion (29). We show that knockdown of either Hsc70 or the ESCRT-I subunit TSG101 compromised the ability of SB203580 to reduce αSyn secretion. However, when we altered the consensus motif for Hsc70 recognition in αSyn by site-directed mutagenesis, we found no effect on the ability of SB203580 to promote degradation of the mutated αSyn, which discounts classical microautophagy as degradation route (29). Importantly though, αSyn distinguishes itself from soluble monomeric client proteins of microautophagy by its propensity to form (templated) oligomers/aggregates, here promoted by either p25α-mediated aggregation or αSyn fibril seeding, and its lipid-binding properties (62). It has elegantly been shown that oligomerization and lipid membrane association alone are sufficient to confer ILV inclusion *via* the ESCRT machinery to otherwise soluble proteins (63). Furthermore, in its propensity as a chaperone, Hsc70 also binds αSyn protein oligomers and aggregates (64, 65) in a “generic” rather than consensus sequence–directed manner and may thus still act as a guiding factor for αSyn to the endosomal membrane. This would also explain why there is a relative enrichment of higher molecular weight αSyn species in secreted exosomes following SB203580 treatment and raises the possibility that other misfolding disease–associated proteins, which share these traits including Hsc70 binding, could be substrates of ESCRT import.

p38MAPK inhibition may increase efficiency of the ESCRT import mechanism itself, or alter transport through the endosomal pathway, such that endosomes have a longer half-life (before fusion with lysosomes) yielding more time for ESCRT function and αSyn import. Along these lines, it is interesting to note that p38MAPK activity through its unloading of Rab5 from early endosomes, where ILV formation commences, positively regulates the traffic through the endosomal pathway (66).

In primary cortical neurons from (h)-αSyn transgenic mice seeded with human αSyn fibrils to induce synucleinopathy, SB203580 had a positive effect on the removal of Ser-129 phosphorylated αSyn, which is enriched in Lewy bodies in PD patient brain (4), and accumulates with disease progression (9, 10). SB203580 decreased p38MAPK phosphorylation (activation), under basal and provoked (lysosomal protease inhibition) conditions, and dose-dependently decreased levels of αSyn p-Ser129 under conditions where macroautophagosomal degradation was blocked. In PC12 cells and primary neurons, accumulated αSyn and αSyn-p-Ser129, respectively, were eliminated under conditions of lysosomal incapacitation by either bafilomycin A1 or protease inhibitors, where the degradation of other substrates such as p62/SQSTM1 was expectedly decreased. We believe this relates to the substantial degradative capacity inherent to late endosomes and the protease sensitivity of αSyn. Late endosomes can readily degrade internalized endocytic or cytosolic substrates (29, 67), and hydrolases are continuously replenished to late endosomes from the biosynthetic pathway. Under conditions where the autophagosomal pathway, including fusion with lysosomes, is compromised, the endosomal pathway may still be operating to completion (17, 68, 69), because of the different fusion factors involved (18, 22). Our experiments with atto-αSyn uptake indicate that mild endolysosomal alkalization following SB203580 treatment is not prohibitive for αSyn degradation presumably by cathepsins (33, 34), which we found elevated by SB203580 treatment. Interestingly, a recent study used FRET-based screening methodology to identify p38MAPK inhibitors as agents that lessen the burden of seeded αSyn aggregation in a cellular reporter system (70).

Collectively, our results indicate that p38MAPK inhibition, by redirecting αSyn degradation from a compromised autophagosomal pathway to late endosomes through ESCRT-mediated import, permits the degradation of substrates even under conditions of partial fusion block of autophagosomes with lysosomes or other autophagolysosomal deficiency. As a correlate, toxic accumulation of autophagosomes is avoided, resulting in diminished cytopathology, and their exocytosis, corresponding to release of αSyn species, is decreased (17). Our results therefore conceptually raise interesting questions for the future as autophagolysosomal deficiency is a recurring pathological theme in neurodegenerative disease (22, 71).

## Experimental procedures

### Antibodies and chemical reagents

Antibodies used included mouse total anti-αSyn monoclonal antibodies (mAbs) (catalog no.: 610787; BD Transduction Laboratories), 4B12 (catalog no.: MA1-90346; Invitrogen), LB509 (catalog no.: sc-58480; Santa Cruz), p-Ser129 αSyn (catalog no.: AB51253; Abcam), and rabbit anti-αSyn pAb (catalog no.: S3062; Sigma); rabbit anti-LC3B (catalog no.: L7543; Sigma or catalog no.: NB600-1364, Novus); anti-p62/SQSTM1 (catalog no.: P0067, Sigma; catalog no.: 51145, Cell Signaling); rabbit anti-LAMP2A pAb (catalog no.: ab18528, Abcam); rabbit anti-LAMP1 pAb (kind gift of Dr Sven Carlsson, Umeå University, Sweden); rat anti-hsc70 mAb (catalog no.: Ab19136, Abcam); rabbit anti-p38MAPKα (catalog no.: 9218, Cell Signaling Technology in Figure 5*A*; catalog no.: SMC-152D from StressMarq Biosciences in Fig. 5*G*), and anti-p38MAPKγ (catalog no.: 2307, Cell Signaling) pAbs; p-p38MAPK was detected by mAb (catalog no.: 4511S, Cell Signaling Technology), and total p38MAPK was detected by rabbit pAb (catalog no.: 9212; Cell Signaling Technology); anti-mouse β-actin (catalog no.: A5441, Sigma), Map2 (catalog no.: M4403, Sigma), and ubiquitin (catalog no.: VU1, Synaptic Systems) mAbs were used. The following chemicals were used: bafilomycin A1 (catalog no.: B1793), leupeptin (catalog no.: L2884), pepstatin A (catalog no.: P5318), SB203580 (catalog no.: S8307), doxycycline (catalog no.: D9891), E64 protease inhibitor (catalog no.: E3132), TCA (catalog no.: T0699), protease (catalog no.; P8340), and phosphatase (catalog no.: P5726) inhibitor were all purchased from Sigma. 3-MA and MG132 were purchased from Calbiochem, and LysoSensor (catalog no.: L-7535) was purchased from Molecular Probes, and Hoechst staining dye solution from Invitrogen (catalog no.: 33342).

### Cell cultures and neuronal differentiation

The rat pheochromocytoma cell line PC12 (American Type Culture Collection) was cultured on collagen-coated culture dishes (catalog no.: 5005-B; Advanced Biomatrix) in Dulbecco's modified Eagle's medium (catalog no.: 6046; Sigma) containing 10% horse serum (catalog no.: 26050-088; Gibco), 5% fetal calf serum (catalog no.: 10270-106; Gibco), and 1% penicillin and streptomycin (P/S) (catalog no.: P0781; Sigma) at 37 °C in 5% CO_2_. Generation of stable cell lines with doxycycline-inducible expression of αSyn and p25α has been described (17). Cells were NGF differentiated for all experiments. In general, cells were seeded at a density of 100,000 cells/cm^2^ in PC12 differentiation medium (1% P/S and 2% horse serum [catalog no.: 26050-088; Gibco]) and 100 ng/ml NGF (catalog no.: 2.5S PM1042; Serotec) for 48 h. Medium was then exchanged with N2 medium (Dulbecco's modified Eagle's medium [catalog no.: 41965; Thermo Fisher Scientific], 1% P/S, and N2 supplement [catalog no.: 17502-048; Gibco]) with NGF, and transgenes were induced by doxycycline (100 ng/ml) with or without SB203580 (or other drugs) for an additional 2 days. Primary neuronal cultures were prepared from mice expressing human wildtype αSyn under the mouse αSyn promoter (51) on day 16 of gestation. Embryos were decapitated, and the brains put into ice-cold Hibernate E (catalog no.: A12476-01; Gibco). Cortices were dissected and dissociated using warm trypsin/EDTA/Hibernate E solution at 37 °C for 15 min. Trypsin solution was removed, and cells resuspended in minimal essential medium (catalog no.: 21430; Gibco) supplemented with GlutaMax (catalog no.: 35050; Gibco), 1% P/S (catalog no.: 15140; Gibco), 10% heat-inactivated horse serum (catalog no.: 16050; Gibco), 0.6 w/v% glucose, 0.22 w/v% sodium bicarbonate, and 5 mM pyruvic acid. Cells were triturated by manual pipetting and seeded at a density of 100,000 cells/cm^2^ on poly-l-lysine (catalog no.: P1399; Sigma)-coated plates. Three to four hours after plating the medium was changed to Neurobasal Plus Medium (catalog no.: A3582901; Gibco) supplemented with B-27 Plus (catalog no.: A3582801; Gibco), and 1% gentamicin. At day *in vitro* 3 (DIV3), 2 μM final concentration of cytosinarabinosid (AraC) (catalog no.: C-1768; Sigma) was added to cultures to prevent proliferation of contaminating glia cells. At DIV5, cells were seeded with fibrillated αSyn at 1 μg/ml final concentration.

### Analysis of pH and endosomal degradation of atto-αSyn

To assess changes in endolysosomal pH, differentiated PC12 cells were incubated in 1 μM Lysosensor Green for 1 h at 37 °C. After a wash, cell fluorescence was measured in a microplate reader (Molecular Devices FLEX station) using wavelengths 488/520 for excitation and emission. Differentiated PC12 cells were incubated with 1 μg/ml atto-conjugated recombinant αSyn for 3 h. Bafilomycin A1 (100 nM) was included as a control and subtracted from sample values for presentation. After incubation, cells were washed once and then flushed off with a pipette for immediate analysis of atto fluorescence in a Beckton Dickinson Accuri C6 flow cytometer using the 488 nm laser line for excitation.

### TCA protein precipitation and LDH assay

Conditioned N2 medium was harvested and centrifuged at 800*g* for 5 min, room temperature (RT), before TCA was added to the supernatant and incubated on ice for 10 min. The protein precipitates were pelleted by centrifugation (16,100*g*, 10 min, 4 °C) and washed 4 to 5 times in ice-cold acetone until the pellet appeared clear white. The pellets were dried at 95 °C for 20 min, dissolved in 2.5× Laemmli buffer, boiled for 20 min at 95 °C, and subsequently processed for Western blotting. Throughout, TCA fractions were loaded relative to the protein concentration of cell lysates to obtain equal loads per lane. An aliquot of the cleared conditioned medium was routinely analyzed for LDH activity by a spectrophotometric method (32) and was similarly normalized to lysate protein concentration for representation.

### Western blotting

Cells were extracted in 1% Triton X-100 (100 mM NaCl, 50 mM Tris–HCl, 1 mM EGTA, 10 mM MgCl_2_, pH 7.2 with phosphatase and protease inhibitors) for 20 min on ice and then for 20 min at RT. Cell lysates were centrifuged (16,100*g*, 5 min, 4 °C), and protein concentration of the lysates was measured with Dc protein assay (Bio-Rad), prior to addition of 5× Laemmli buffer and loading of equivalent protein quantities on SDS-polyacrylamide gels for transfer and Western blotting using horseradish peroxidase–conjugated pAbs goat antimouse (catalog no.: P0447; Dako) and swine anti-rabbit (catalog no.: P0399; Dako) for chemiluminescent horseradish peroxidase detection (Millipore). For Western blot experiments, normalization to actin was not performed routinely, as we found that actin levels were themselves altered by some of the treatments and experimental conditions used. Instead, consistency of protein load, electrophoresis, and transfer was verified by Coomassie blue staining of the gel after transfer and Ponceau S staining of the membrane after transfer. Should these techniques reveal a problem, the whole gel was run again. Primary neurons were lysed for 1 h in radioimmunoprecipitation assay buffer (catalog no.: R0278; Merck) supplemented with benzonase (catalog no.: E1014; Sigma), PhosSTOP, and cOmplete (Roche) at 4 °C. Samples were boiled for 5 min and separated on NuPAGE Novex 4 to 12% Bis–Tris Gels (catalog no.: NP0323; Thermo Fisher Scientific) in NuPAGE MES SDS Running Buffer (catalog no.: NP0002; Thermo Fisher Scientific). A prestained Chameleon Duo marker (catalog no.: 928-6000; LI-COR) was used as reference. Gels were blotted on to 0.45 μm polyvinylidene difluoride membranes (catalog no.: IPFL00010; Merck) for 2 h at 25 V. Membranes were boiled for 5 min in PBS, blocked in Odyssey Blocking buffer (catalog no.: 927-4000; LI-COR), and incubated with primary antibodies overnight at 4 °C. Membranes were then washed, incubated with secondary antibodies (goat antimouse Alexa 680 nm) (catalog no.: A21058; Thermo Fisher Scientific) and goat anti-rabbit IRDye 800 nm (catalog no.: 926-32211; LI-COR) for 1 h in blocking buffer supplemented with 0.02% SDS and 0.1% Tween-20, and scanned with an infrared Odyssey CLx scanner (LI-COR Odyssey Biosystems).

### Immunofluorescence

PC12 cells were fixed in a phosphate buffer containing 2% PFA, pH 7.4. Cells were permeabilized and blocked in 5% normal goat serum with 0.2% saponin in PBS for 15 min, followed by 45 min incubation with primary antibodies. After PBS wash, secondary antibodies Alexa488-, 568-, or 647-conjugated goat anti-mouse, anti-rat, or anti-rabbit as specified were applied for 45 min. Images were acquired with a Zeiss LSM510 confocal laser scanning microscope with a C-Apochromat 63×, 1.4 numerical aperture oil immersion objective. Confocal sections of 1 to 1.2 μm were collected and saved as 1024 × 1024-pixel images at 12 bit resolution before quantification or compilation in Adobe Photoshop CS2, version 9.0.2, Zeiss LSM image browser, or ImageJ (using the colocalization algorithm http://uhnresearch.ca/wcif/ImageJ to obtain Pearson coefficients). Primary neurons were fixed in 4% PFA for 20 min at RT followed by permeabilization with 0.1% Triton X-100 (Sigma–Aldrich). Primary antibodies were diluted in PBS and incubated with cells overnight at 4 °C followed by washing. Secondary Alexa488- or Alexa568-conjugated donkey anti-mouse (A21202) and donkey anti-rabbit (A10042) antibodies and Hoechst (33342; Invitrogen) were incubated with cells for 1 to 2 h at RT. The cells were kept in PBS for imaging using a Cellomics Array Scan V High Content Screening Reader (Thermo Fisher Scientific) or mounted for imaging with a spinning disk confocal microscope (PerkinElmer UltraVIEW VoX 3D Live Cell Imaging System). Images were analyzed using Volocity software.

### Cell transduction and vectors

Lentivirus production and vectors have been described previously (17, 72). The ^95^VKKAA mutant was introduced into vector pLOX TW αSyn using standard procedures and verified by sequencing. The following lentivectors with shRNA coding 19- or 21-mers were selected for use: p38MAPK (pGIPZ vectors from Open Biosystems; -α, TATACTTCAGCCCTCGGAG; -γ1, TTCAAGATGACCTCTGGTG; -γ2, TCTGATACACAAGAAACTG), Hsc70 (pLKO1 vectors from SIGMA Mission; GCTCGATTTGAGGAGTTGAAT), LAMP2a (pGIPZ; CTGAACAACAGCCAAATTA), TSG101 (pLKO1; GCTATTGAAGACACTATCTTT), and Ndfip1 (pLKO1; GTTCGGAAGATGCCAGAAACT). Cells were transduced for 48 h before doxycycline induction of αSyn and/or p25α for a further 48 h of culture before sampling and medium collection. Virus dose was in each case titrated to obtain the highest possible knockdown of the gene of interest without cytotoxicity, crucial for analysis of secreted αSyn, as measured by LDH release. For siRNA treatment, primary neurons were treated with 1 μM siRNA with 2 μM AraC DIV3. About 125 ng of αSyn fibrils were added from DIV7 to DIV13 in a 50% medium change. siRNA was ordered from Accell, with a Nontargeting Control Pool siRNA as control (D-001910-10) and a pool of two sets targeting SNCA with the following sequences. (1) sense: GGGUGUUCUCUAUGUAGGCUU and antisense: 5′-PGCCUACAUAGAGAACACCCUU (2) sense: GAGCAAGUGACAAAUGUUGUU and antisense: 5′-PCAACAUUUGUCACUUGCUCUU.

### Sucrose gradient fractionation

PC12 cells were homogenized by successive aspiration of cell suspension in ice-cold hypotonic buffer (75 mM NaCl, 10 mM Hepes, 170 mM sucrose, 1 mM MgCl_2_, 1 mM EGTA, pH 7.4) into 23G and then 27G syringe needles. The homogenate was centrifuged at 800*g* and then 10,000*g*, to remove unbroken cells and mitochondria, respectively, and then layered on top of a 15 to 40% sucrose gradient and centrifuged at 80,000*g* overnight in a swinging bucket SW40Ti rotor. Fractions were collected from the bottom with a peristaltic pump, and aliquots were analyzed by Western blotting.

### Exosome preparation

Conditioned medium from a 10 cm petri dish was collected, passed through a 0.22 μm filter, and then centrifuged at 10,000*g* to remove debris prior to ultracentrifugation at 100,000*g* for 1 h in a SW40Ti swinging bucket rotor to pellet exosomes. The pellet was washed once in PBS, recentrifuged, and then lysed for analysis.

### Fibrillar αSyn seeding material

αSyn fibrils were made according to the protocol of the Virginia Lee laboratory (73). In short, human recombinant αSyn was expressed in human embryonic kidney 293 cells, purified, and concentrated by centrifugation at 1000 rpm before fibrillation for 5 days at 37 °C. Fibrils were then sonicated (Q800R2; Qsonica: 20% power for 10 min) and subjected to quality control by bacterial growth and endotoxin tests, size determination by light scattering, thioflavin T staining, and electron microscopy.

### Cellomics

Cellomics Array Scan V High Content Screening Reader (Thermo Fisher Scientific) was used to assess immuno-stained neuronal populations. About 40 to 60 pictures per 96 wells were taken and subjected to image analysis applying a Cellomics algorithm. This algorithm quantitates the area and intensity of marker proteins visualized by immunocytochemistry in a specified region around the cell nuclei. Furthermore, the algorithm was set up to count the number of viable cells per well by gating out condensed and small nuclei. Note that the number of viable cells did not differ significantly between treatments (Fig. 8*C*).

### Statistics

Raw data were analyzed by ordinary one-way ANOVA with the post hoc Sidak’s or Dunnett’s correction for multiple comparisons. Normalized data, which do not fulfill the criteria of equal variance, were analyzed by nonparametric Kruskal–Wallis tests with post hoc Dunn’s correction for multiple comparisons or one-sample *t* test where appropriate. Significance level was set as follows: *p* ≥ 0.05 (not significant, ns), *p* ≤ 0.05 (∗), *p* ≤ 0.01 (∗∗), *p* ≤ 0.001 (∗∗∗), and *p* ≤ 0.0001 (∗∗∗∗), and N refers to the number of individual experiments. The 95% confidence intervals on percentage changes were calculated using the formula: (%chg_AtoB_) ± 1.96 ∗ SE(%chg_AtoB_), with SE(%chg_AtoB_) = |B/A| ∗ √[(SE_B_^2^/SE_A_^2^) + (SE_A_^2^/SE_B_^2^)] ∗ 100.

## Data availability

All data are contained within the article.

## Supporting information

This article contains supporting information.

## Conflict of interest

The authors declare that they have no conflicts of interest with the contents of this article.
