# Supplementary material for: α-synuclein buildup is alleviated via ESCRT-dependent endosomal degradation brought about by p38MAPK inhibition in cells expressing p25α
Source: J Biol Chem. 2022 Sep 24;298(11):102531. doi: 10.1016/j.jbc.2022.102531 (PMC9637583; doi:10.1016/j.jbc.2022.102531)
Supplement: Supporting information [file mmc1.docx]

**α-synuclein build-up is alleviated via ESCRT-dependent endosomal degradation brought about by p38MAPK inhibition in cells expressing p25α**

**SUPPLEMENTARY INFORMATION**

**Quantitative real-time polymerase chain reaction (qPCR)**

Cell lysis, genomic DNA degradation and reverse transcription were performed according to the instructions of the Ambion Fast SYBR Green Cells-to-CT Kit (ThermoFisher). qPCR reactions were run on 0,6x diluted cDNA, 0,3 µM primers and 2x diluted SsoFast EvaGreen Supermix (Bio-Rad) on CFX384 Real-Time System, C1000 Touch Thermal Cycler (Bio-Rad). CT-values were normalized to the geometric mean of the three housekeeping genes (GPI1, SNRPD3, and HPRT) using the delta-delta Ct-method. Custom made SNCA primers were ordered from Primer Design; Forward: CAAGTGACAAATGTTGGAGGAG, Reverse: CTGCTGCAATGCTCCCT.

**Cisbio Homogenous Time-Resolved Fluorescence**

The amount of p-Ser129 and aggregated α-synuclein in cell lysates were measured by Homogenous Time Resolved Fluorescence commercial kits 6FSYNPEG and 6FASYPEG respectively (Cisbio), following the manufacturer’s instructions. In brief, cells were washed and lysed for 1 hour at 4ºC in lysing buffer (CFUS000, Cisbio) supplemented with blocking reagent and Benzonase® Nuclease (E1014, Sigma). The two kit antibodies d2 and Tb Cryptate were diluted in detection buffer and mixed 1:1. 9μl of cell lysates and 9μl of antibody mix were added to flat-bottom 384-well microplates (784075, Greiner) with the following controls: buffer control to normalize samples to; quality controls with PBS instead of kit antibodies (blanks); cryptate control with only the Tb antibody for specificity; positive control with phosphorylated or aggregated α-synuclein protein supplied by the kit. Plates were sealed and spun down at 1000g for 1min and read after 3 hours in the dark on a PHERA star plate reader (BMG Labtech).
