# Supplementary material for: α-synuclein buildup is alleviated via ESCRT-dependent endosomal degradation brought about by p38MAPK inhibition in cells expressing p25α
Source: J Biol Chem. 2022 Sep 24;298(11):102531. doi: 10.1016/j.jbc.2022.102531 (PMC9637583; doi:10.1016/j.jbc.2022.102531)
Supplement: Supplemental Figures S1–S6 legends [file mmc2.docx]

**α-synuclein build-up is alleviated via ESCRT-dependent endosomal degradation brought about by p38MAPK inhibition in cells expressing p25α**

**SUPPLEMENTARY FIGURE LEGENDS**

**Supplementary Figure 1. SB203580 alters lysosomal pH and proteases**

A) NGF-differentiated PC12 cells expressing αSyn alone or αSyn/p25α were treated with SB203580 for two days, and then lysed for western blot analysis of pro-cathepsin D (Ordinary one-way ANOVA, N=3). B) A full-length western blot to show the effect of p25α expression and SB203580 on pro-cathepsin D and mature and cleaved cathepsin D. Molecular weight markers indicated. C) NGF-differentiated PC12 cells expressing αSyn alone or together with p25α were treated with SB203580 as indicated for two days and then incubated with Lysosensor for 30 minutes before cell detachment and immediate flow cytometric analysis (mean ±SEM, ANOVA, N=3). D) Representative images of Lysosensor fluorescence in NGF-differentiated PC12 αSyn/p25α cells treated with 1 μM SB203580 or bafilomycin A1 (100 nM) as control of alkalinisation. Bar, 10 μm.

**Supplementary Figure 2. SB203580 effect on αSyn turnover is independent of macroautophagy**

A) NGF-differentiated PC12-αSyn/p25α cells were treated with indicated concentrations of SB203580 for two days before western blot analysis of p62 and LC3-I/II in the lysate fraction (Kruskal Wallis test (KW), N=3-4). B) NGF-differentiated PC12-αSyn/p25α cells were treated with doxycycline and 1 μM SB203580 w/wo autophagy inhibitor 3-MA at concentrations indicated (μM) for two days before western blot analysis of αSyn, p62, and LC3. B-E) Quantitation of experiments above for C) αSyn in lysate or D) medium fraction, and E) p62, and F) LC3-II in the lysate fraction (mean ±SEM, Kruskal-Wallis tests, N=3). G) Indirect immunofluorescence to localize αSyn (LB509 mAb) relative to LAMP1- or LC3-positive compartments in control or SB203580-treated PC12-αSyn/p25α cells. Note that SB203580 treatment causes a decreased colocalization of αSyn with LC3, but an increased colocalization with LAMP1. The images are representative of two independent experiments. Bars, 10 μm.

**Supplementary Figure 3. SB203580 effect on αSyn turnover is independent of proteasomal activity**

NGF-differentiated PC12 cells expressing αSyn/p25α were treated with proteasomal inhibitor MG-132 at concentrations indicated with or without 1 μM SB203580 for 48 hours and then analyzed for αSyn in the A) conditioned medium or B) lysate fraction by western blotting (mean ±SEM, Kruskal-Wallis tests, N=3). All lanes shown in either A) or B) are from the same gel, but their order has been inverted. C) Lysate fractions were also western blotted with two different anti-ubiquitin antibodies (VU1 or K48 antibodies) to show effect of MG132 treatment, note the increased immunoreactivity for ubiquitin with increasing MG132 concentration. Molecular weight markers indicated.

**Supplementary Figure 4. Expression of p38MAPK isoforms in PC12 and SHSY-5Y cells**

A) NGF-differentiated PC12 cells or BDNF-differentiated SHSY-5Y cells expressing αSyn alone or together with p25α were lysed and analysed for expression of p38MAPK isoforms by western blotting as indicated. The shown blot is representative of two independent trials. Note how p25α expression for each p38MAPK isoform decreases their protein level.

**Supplementary Figure 5. Eukaryotic αSyn fibrils seed endogenous synucleinopathy in primary neurons overexpressing wild type human αSyn**

A) Human αSyn fibrils analyzed by Nanoparticle Tracking before (green) and after (orange) sonication, particle counts are binned and plotted against bin center (bin size =1nm). Representative electron micrographs of fibrils before (B) and after (C) sonication, scale bar =500nm. D) Schematic overview of the primary neuronal culture treated with αSyn siRNA after 3 days in vitro (DIV), αSyn fibrils on DIV7 and scored for p-ser129 αSyn signal on DIV13. E) Pictures from the high-content image analysis using a Cellomics array scanner showing an example of p-ser129 staining, scoring by the algorithm, and an overlay of nuclear (Hoechst), p-Ser129 and MAP2 neuronal staining in untreated neurons and neurons treated with seed and siRNA targeting either the αSyn gene (siSNCA) or with a non-targeting control (siCtr). Bars, 40 μm. F) Quantification of p-ser129 αSyn staining by the Cellomics Array scanner on fixed and stained cells (Ordinary one-way ANOVA, mean ±SEM, N=2 biological replicates). G) A concentration curve of 5-80 ng/mL α-synuclein fibrils was added at DIV5 to the primary mouse cortical neurons for 1 week before cell lysates were harvested and scored for αSyn p-ser129 and aggregation using the appropriate Cisbio Homogenous Time-Resolved Fluorescence assays in parallel. Graphs show mean±SD.

**Supplementary Figure 6. Effect of SB203580 addition post-seeding to primary cortical neurons**

A) Primary cortical neurons were treated with αSyn fibrils for 5 days, before SB203580 was added to cultures for a further 2 days. B) Cells were fixed and processed for indirect immunostaining of p-Ser129 αSyn and subsequent quantitation by Cellomics Array scanner. Graphs show quantitation of B) p-Ser129 αSyn staining and C) live cells (Ordinary one-way ANOVA, mean ±SEM, N=2 biological replicates).
