# Supplementary figures and images for: α-synuclein buildup is alleviated via ESCRT-dependent endosomal degradation brought about by p38MAPK inhibition in cells expressing p25α
Source: J Biol Chem. 2022 Sep 24;298(11):102531. doi: 10.1016/j.jbc.2022.102531 (PMC9637583; doi:10.1016/j.jbc.2022.102531)

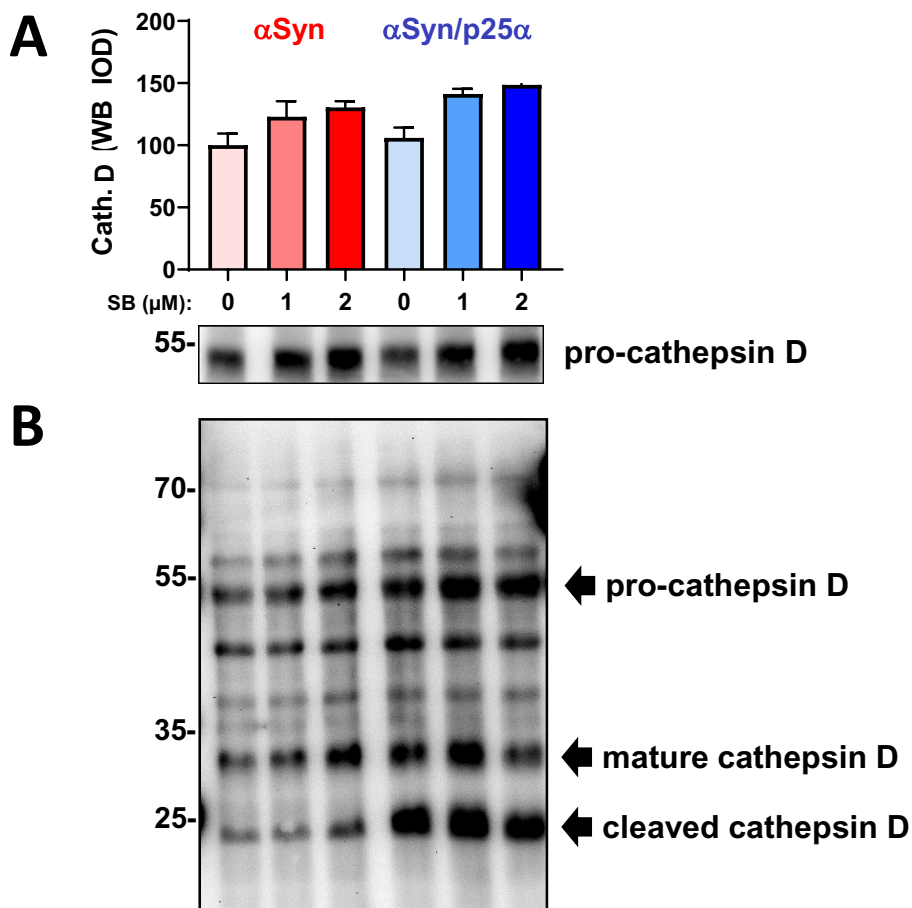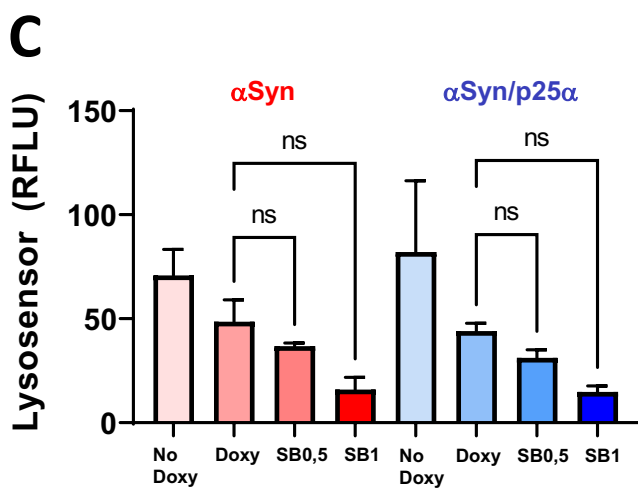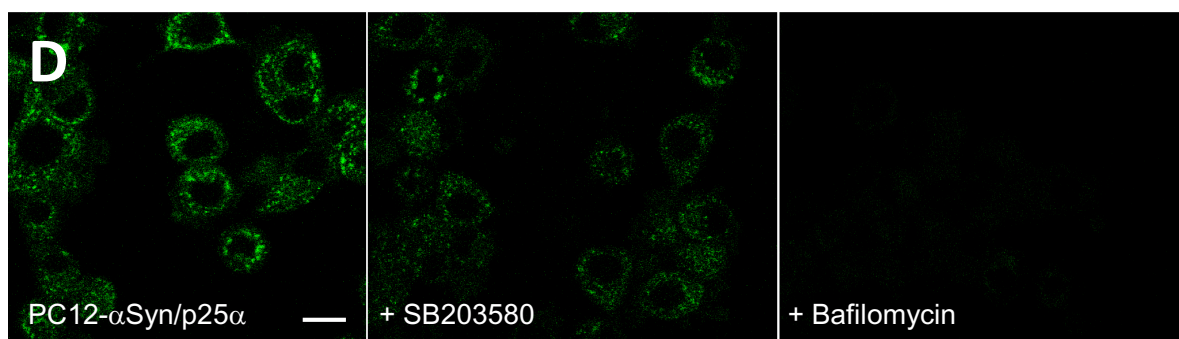

Suppl. Figure 1

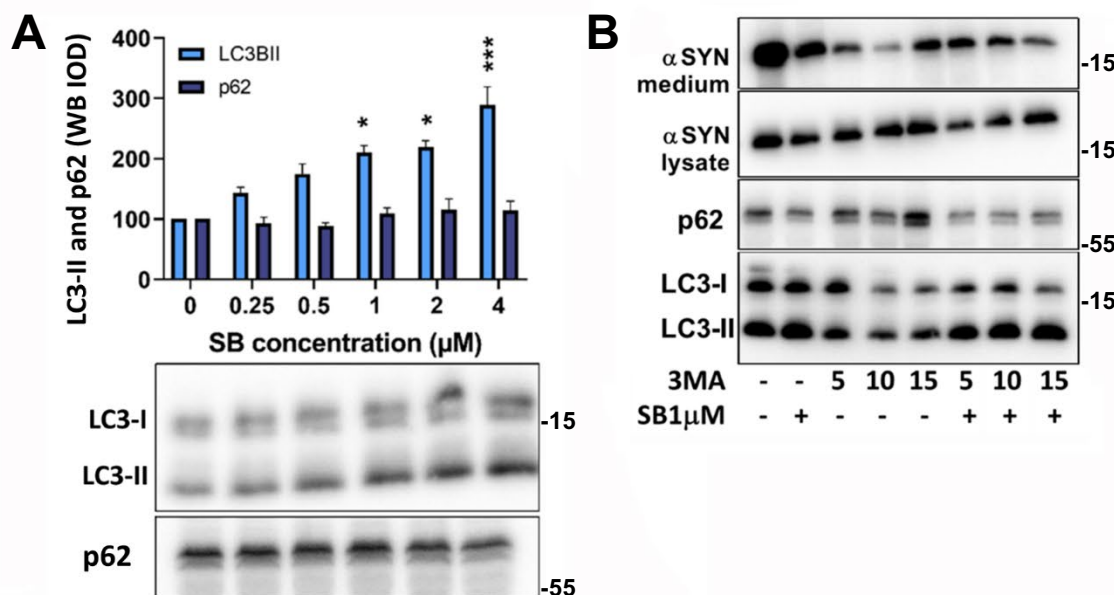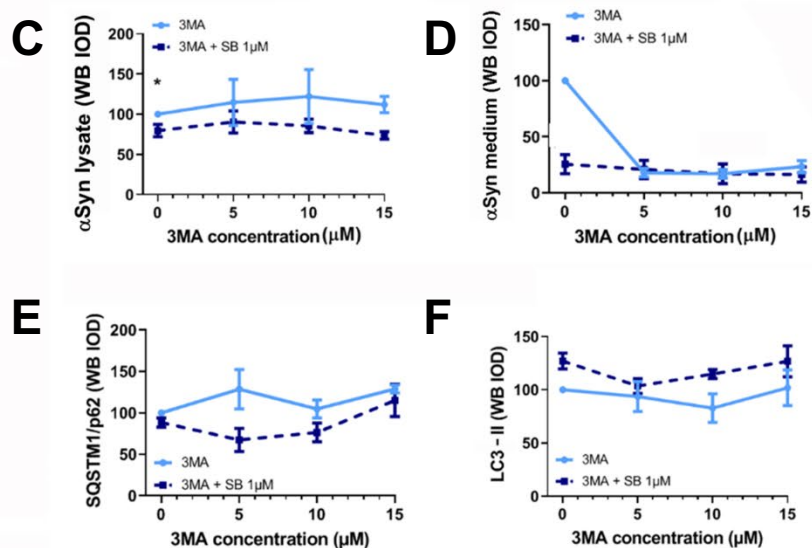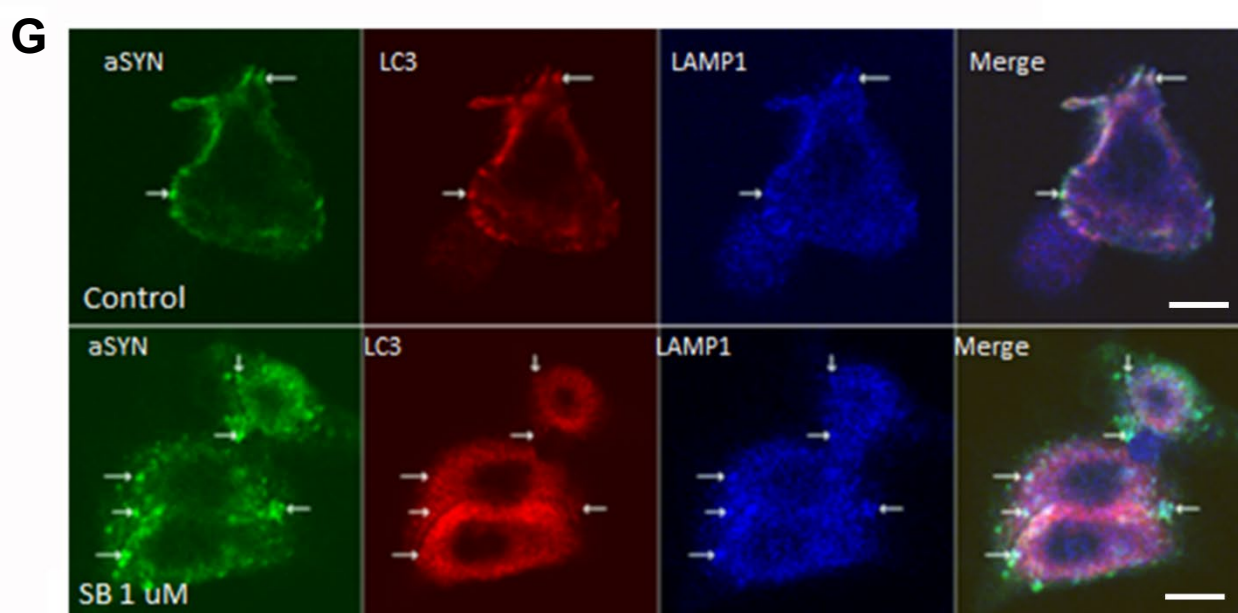

Suppl. Figure 2

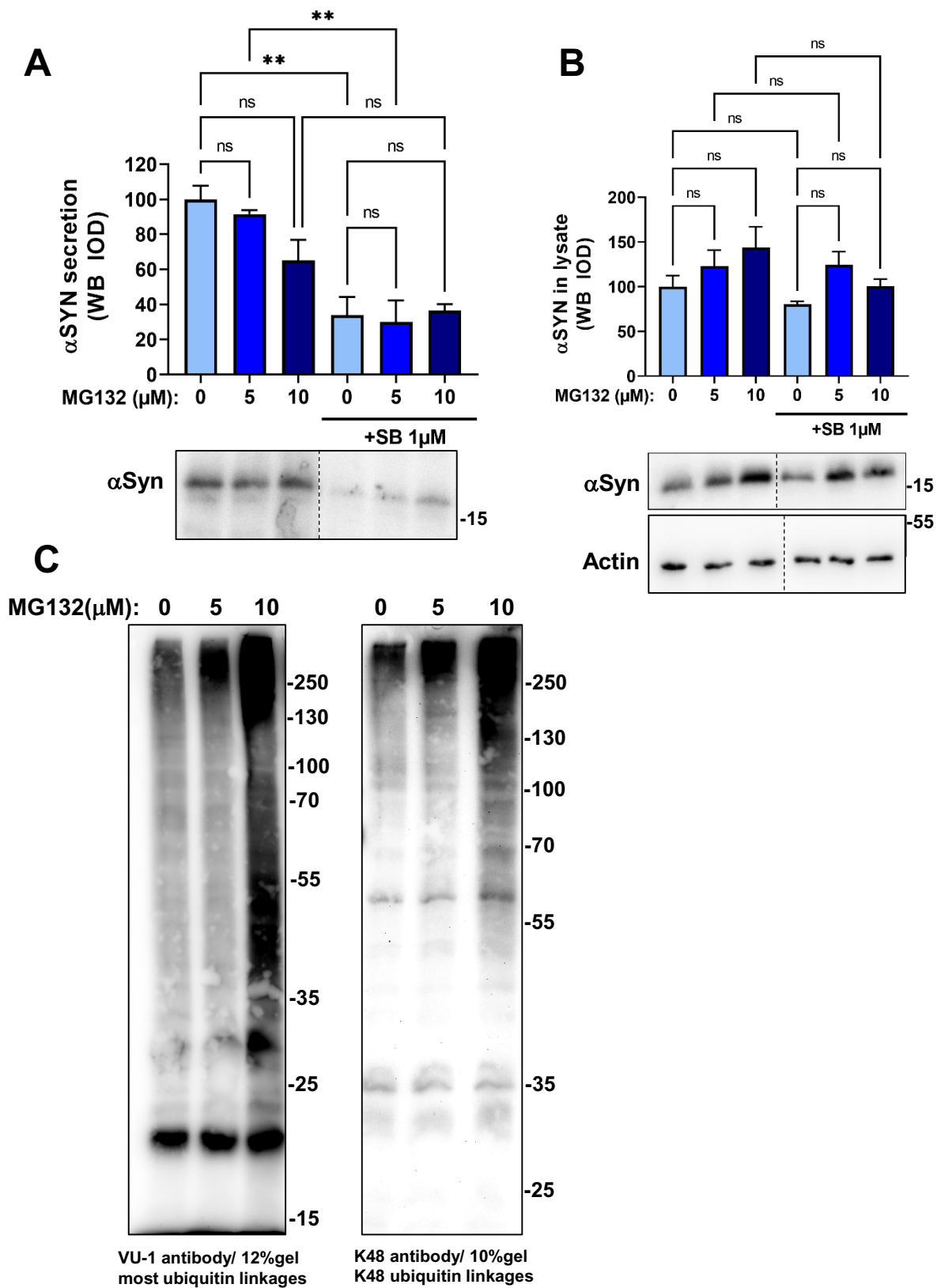

Suppl. Figure 3

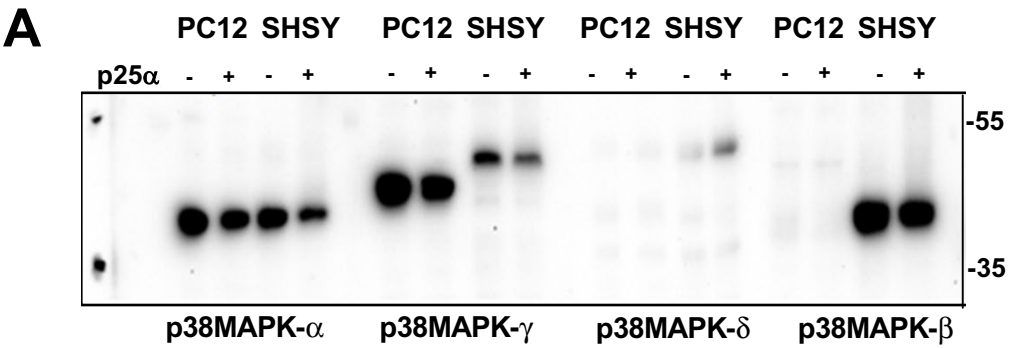

Suppl. Figure 4

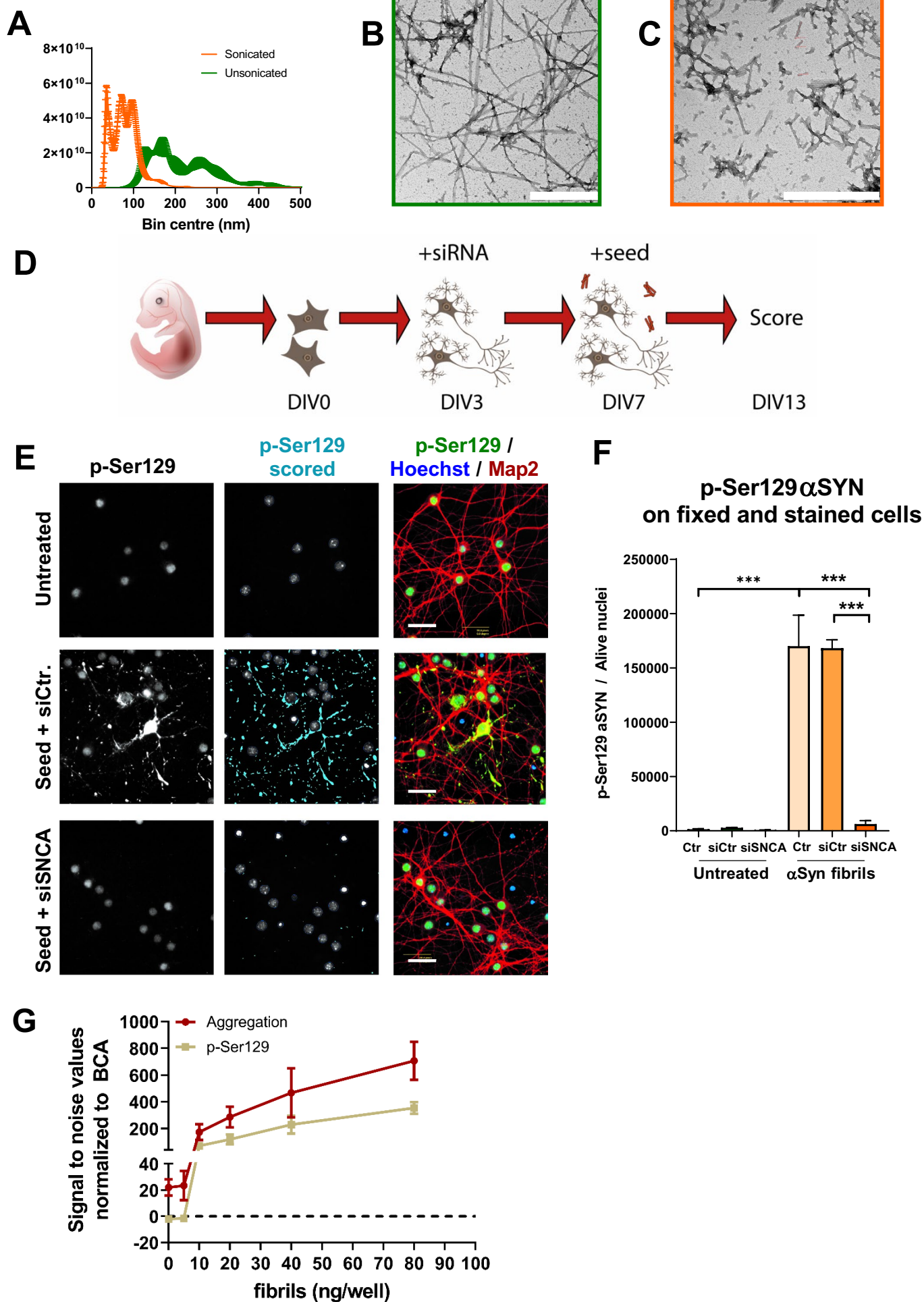

Suppl. Figure 5

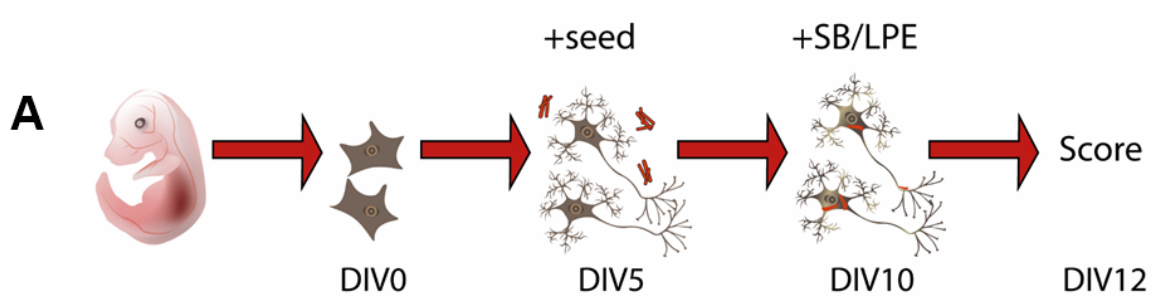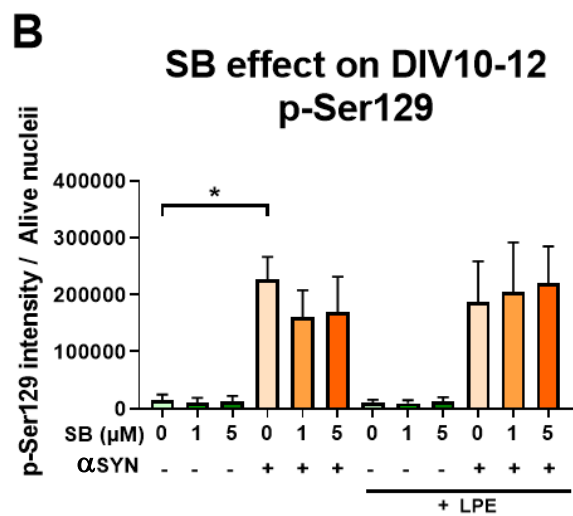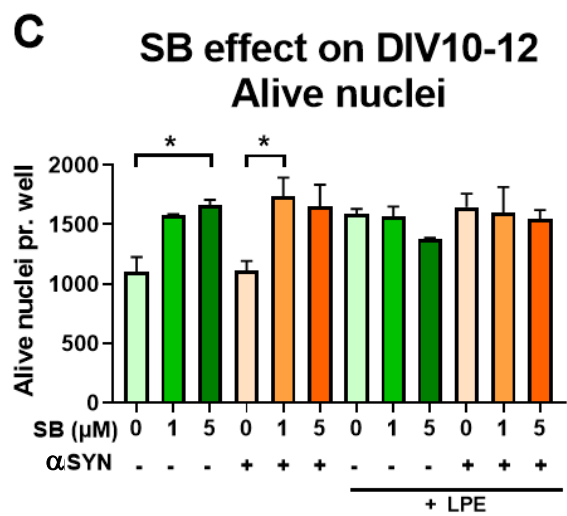

Suppl. Figure 6

Supplement: Supplemental Figures S1–S6 [file mmc3.pdf]
